# Design of efficient non-doped blue emitters: toward the improvement of charge transport†

**DOI:** 10.1039/c9ra04918e

**Published:** 2019-09-04

**Authors:** Sunwoo Kang, Jong Hun Moon, Taekyung Kim, Jin Yong Lee

**Affiliations:** Display Research Center, Samsung Display Co. 1 Giheung-gu Gyunggi South Korea swkang821221@gmail.com; Department of Chemistry, Sungkyunkwan University Suwon 16419 South Korea jinylee@skku.edu; Department of Materials Science and Engineering, Hongik University Sejongsi 30016 South Korea taekyung.kim@hongik.ac.kr

## Abstract

Charge transport and electronic transition properties of a series of newly designed anthracene-based non-doped blue emitters were investigated by density functional theory calculations. For a highly efficient non-doped device, Cz3PhAn-based emitters were designed to suppress the hole and electron reorganization energies required for structural relaxation with respect to the changes of charged states. As a result, the hole hopping rates of triphenylamine (TPA) and phenylbenzimidazole (PBI) substituted Cz3PhAn derivatives (1, 4, and 5–7) were tremendously enhanced as compared to that of Cz3PhAn due to the suppression of the reorganization energy of holes, *λ*_h_. Moreover, 1 and 4 emitters showed almost identical hopping rates of holes and electrons, which can possibly lead to a perfect charge balance and high efficiency. The photo-physical properties showed that the emission energy of all 1–10 emitters is in 439–473 nm range. It is expected that our rational design strategy can help develop non-doped blue fluorescent emitters for high efficiency.

## Abstract 2

Non-doped blue emitters are proposed for efficient fluorescence devices with high singlet exciton yield induced by high triplet–triplet fusion.

## Graphical abstract

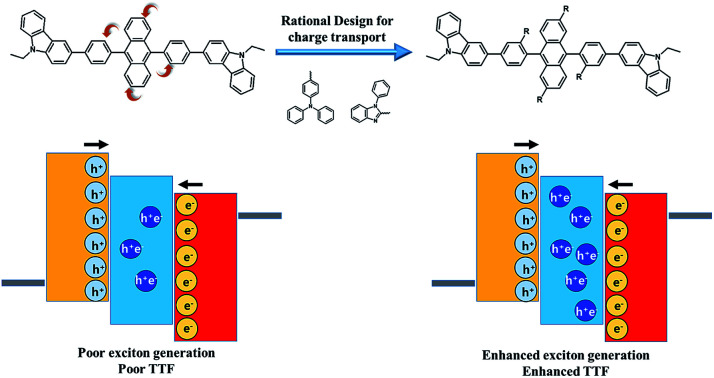

## 1. Introduction

**Fig. 1:**
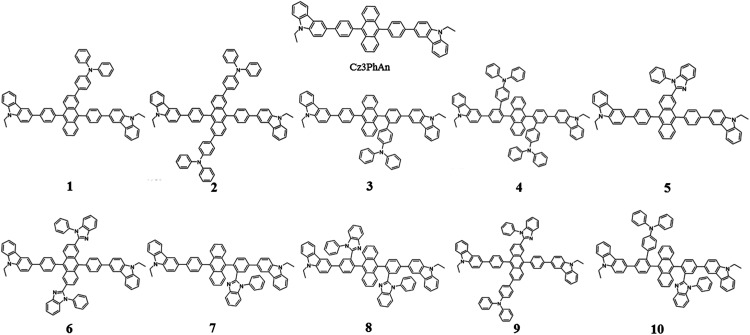
The 2D molecular structures of Cz3PhAn and their derivatives.

Since the first invention of self-emitting devices by Tang *et al.*,^1^ solid state organic light emitting devices have been received tremendous attention in the display industry owing to their wide applications in high quality full-color displays and mechanical flexibility.^2–5^ To achieve high efficiency and long lifetime, many attempts have been made utilizing multi-layered organic light emitting diodes (OLEDs).^6,7^ In order to achieve high efficiency and long lifetime simultaneously, good charge balance and high internal quantum efficiency (IQE) are essential, resulting in multi-functional materials in multi-layered devices.^8^ It is also required to realize a simple-structured OLED to simplify the evaporation processes and reduce the costs.^9–13^ To achieve a simple-structured device, we have to develop the multifunctional materials having good carrier transport and emission properties. Previously, Chang *et al.* reported a series of carbazole-substituted anthracene derivatives for non-doped blue emission (9,10-bis[4-(9-ethyl-9*H*-carbazole-3-yl)phenyl]anthracene = Cz3PhAn).^14^ The Cz3PhAn emitter showed good chemical stability, color purity, and thermal stability in the experiment. In addition, 5.84% of the maximum external quantum efficiency (EQE) was measured in non-doped blue fluorescent OLEDs, in which triplet-to-singlet conversion *via* triplet–triplet fusion (TTF) is believed to play a significant role. One of the most practical solutions to achieve high efficiency is to maximize a TTF-induced singlet yield of anthracene-based non-doped emitters. In order to achieve high TTF-induced singlet yield, it is critical to realize a high triplet–triplet collision rate and an intrinsic triplet-to-singlet conversion rate.^15–18^ Since TTF is a collision process, a high triplet–triplet collision probability strongly depends on the population of triplet excitons in the emitting layer and their spatial distribution.^19–21^ These two factors can be managed by hole and electron mobilities in the emitting layer and interface barriers between neighboring layers. The intrinsic triplet-to singlet conversion is strongly affected by the alignment of energy levels of S_1_, T_1_, and T_2_ of emitting materials. In addition, the intrinsic triplet-to-singlet conversion rate can also be affected by temperature-independent reverse intersystem crossing (RISC) rate. RISC is, in turn, strongly affected by three critical factors; spin–orbit coupling (SOC), Δ*E*(T_2_–S_1_), and vibronic coupling (〈S_1_|*Ĥ*_vib_|T_2_〉).^22–24^ With an assumption of 25% of singlet formation *via* hole–electron recombination, the TTF-induced singlet yield is categorized to be 7.5, 15, and 37.5%, depending on the relative position of S_1_ and T_1_ levels,^16,25^ which leads 32.5, 40.0, and 62.5% of IQEs. To our knowledge, no deep blue fluorescent anthracene-based material with the TTF-induced singlet yield over 15% has been reported. Thus, maximum IQE of TTF-type blue fluorescent anthracene-based materials used in commercial OLED displays is limited up to 40%. To our knowledge, it is extremely difficult to independently control each of three factors (SOC, Δ*E*(T_2_–S_1_), and vibronic coupling). In other words, three key parameters cannot be simultaneously improved while maintaining inherent transition properties. As referred to previous reports, the balanced charge carrier concentration can efficiently enhance the generation of excitons, which lead to increased concentration of triplet excitons. Therefore, increasing the number of triplet excitons can give a better opportunity for the generation of singlet exciton *via* bimolecular annihilation as called triplet–triplet fusion (TTF). In 2014, Zhou *et al.*, reported highly efficient OLED based on TTF fusion.^26^ They noted that a device with better charge balance can guarantee the excellent device efficiency compared with others. They noted that a device with better charge balance can guarantee the excellent device efficiency compared with others. In addition, Chiang *et al.* also reported that increasing mobility can provide highly efficient blue fluorescent device *via* triplet–triplet fusion.^16^ Therefore, we believe that the modification of charge transport properties to manage the charge balance and the spatial density of triplet excitons for high triplet collision probability might be a unique solution to practically enhance the efficiency blue fluorescent OLEDs. According to the Marcus formalism, charge carrier hopping rate strongly depends on the transfer integral and reorganization energy. However, the charge transfer in anthracene-based non-doped blue emitters might be sufficiently prohibited by the presence of vertical alignment between bridging phenyl and anthracene fragment. Therefore, non-doped emitters should be rationally designed to minimize the reorganization energy through the analysis of vibration-coupled relaxation energy. As aforementioned, the improvement of charge carrier mobility of anthracene based non-doped emitters can enhance the device efficiency due to the increase of TTF. Furthermore, in case of anthracene based non-doped emitter, the charge carrier mobility might be easily modulated by reducing the reorganization energy. From this perspective, we have rationally analyzed the structural variations of Cz3PhAn*via* redox reaction. As a result, we have adopted the TPA and BPI substituents to specific position where the vibration modes are expected to highly contribute the reorganization energy. In this report, we introduce a series of Cz3PhAn derivatives that were designed based on the aforementioned theory and investigate their electronic, charge transport, and photo-physical properties by using density functional theory and time-dependent density functional theory simulations. We expect that our designed emitters based on modulation of charge transport property can show excellent device performance *via* enhanced TTF phenomena in OLED device as compared Cz3PhAn (Fig. 1).

## 2. Theory and computation

We carried out the density functional theory (DFT) calculations for Cz3PhAn derivatives using a nonlocal functional of CAM-B3LYP^27^ and double zeta potential basis set with one polarization function (6-31G*) implemented in a suite of Gaussian 09 program.^28^ The optimized structures of ground and singlet 1^st^ excited states were processed without symmetry constrains (*C*_1_). Moreover, the polarized continuum model^29^ was considered to obtain reliable optimized structures of 1^st^ excited state in dichloromethane medium due to the consistency with experiment. However, the CAM-B3LYP functional does not properly describe the electronic properties of Cz3PhAn. To provide quantitatively and qualitatively reliable simulation results, we have additionally carried out single-point energy calculations with B3LYP/6-311G* level of theory due to the consistency with experiment (see Table S1†). The field-independent carrier mobility at molecular level was computed based on the Marcus theory as the following equation.^30^1
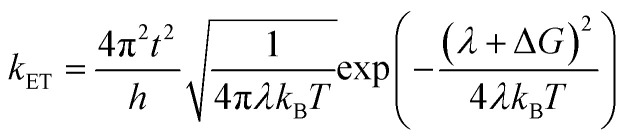
where *t*, *λ*, *k*_B_, *h*, and *T* are defined as transfer integral, reorganization energy, Boltzmann constant, Planck constant, and temperature, respectively. The carrier hopping rate essentially depends on two significant variables; reorganization energy (*λ*) and transfer integral (*t*). The transfer integral of hole (*t*_h_) and electron (*t*_e_) in dimer structures should be considered through the energy splitting due to inter-molecular polarization effect. Thus, transfer integral values were derived by following equation.^31^2
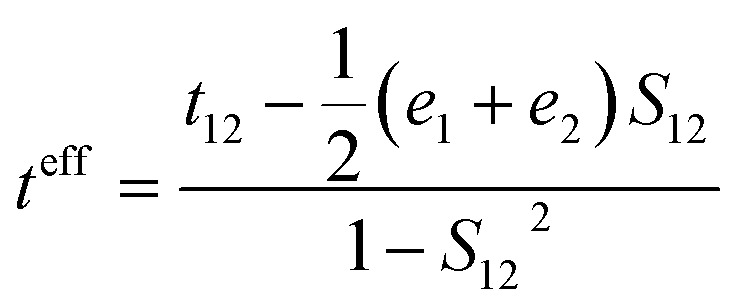
where *t*_12_, *e*_1_, and *e*_2_ are matrix element of system Hamiltonian. *S*_12_ is the spatial electron density overlap integral of highest occupied molecular orbital (HOMO) and lowest unoccupied molecular orbital (LUMO). The inner-reorganization energies of hole and electron can be obtained from the structural relaxation energies *via* oxidative and reductive electrochemical reactions. The equations can be expressed by

3 *λ*_h_ = (*λ*^cation^_neutral_ − *λ*_cation_) + (*λ*^neutral^_cation_ − *λ*_neutral_)

4 *λ*_e_ = (*λ*^anion^_neutral_ − *λ*_anion_) + (*λ*^neutral^_anion_ − *λ*_neutral_)

The vibration coupled relaxation energy *via* redox reaction was analyzed in terms of Huang–Rhys factor, which can be used to quantitatively interpret the energetic contribution of structural changes depending on oxidation and reduction process. It can be expressed as5
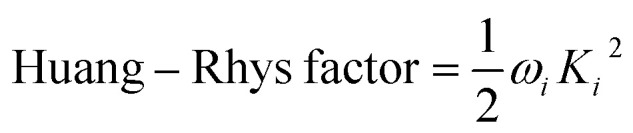
where *K*_*i*_ is the shift vector and *ω*_*i*_ is the vibrational frequency of *i*-th normal vibrational mode. The *K*_*i*_ can be defined as dimensionless displacement vector corresponding to changes in geometries between initial and final state of *i*-th normal vibrational mode.^32–34^ The radiative lifetimes have been generally calculated by Einstein transition probability equation according to the following equation.^35,36^6
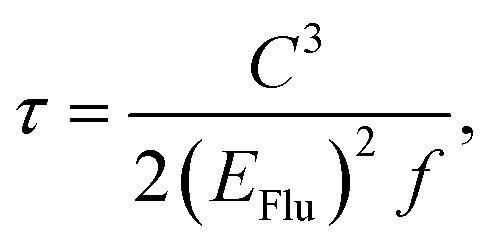
where *C*, *E*_Flu_, and *f* represent the speed of light, singlet emission energy, and oscillation strength, respectively.

## 3. Results and discussions

### 3.1. Analysis of Cz3PhAn

**Fig. 2:**
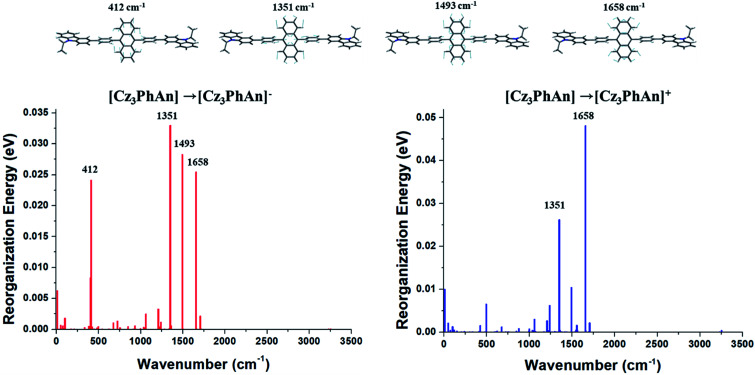
Huang–Rhys factors of Cz3PhAn for charge transitions.

The contributions of vibration modes on hole and electron reorganization energies of Cz3PhAn were analyzed in terms of Huang–Rhys factor. As can be seen in Fig. 2, four vibration modes are clearly distinct and must have high impact on the reorganization energies. The vibration modes can be assigned in-plane C–H bending modes at anthracene and bridging phenyl fragments. In addition, it is noticed that the hole reorganization energy (*λ*_h_) is slightly higher than electron reorganization energy (*λ*_e_). This result implies that electrons in Cz3PhAn may potentially have higher mobility than holes. Furthermore, charge imbalance in non-doped blue emitter may interrupt the efficient exciton conversion into visible photons due to exciton quenching with unpaired charges, called exciton–polaron quenching.^37–39^ It implies that hole carrier mobility should be carefully optimized in order to achieve efficient exciton generation under the good charge balance and high triplet–triplet collision probability. Concerning the analyses of Huang–Rhys factors and synthetic accessibility, the triphenylamine (TPA) and phenylbenzimidazole (PBI) moieties are substituted at 2 and 6 positions of the anthracene fragment and *ortho*-position of the bridging phenyl fragment. As a result, potential candidate materials (1–10) were newly designed and detail analyses of their electronic, charge transport, and photo-physical property are described in the following sections.

### 3.2. Geometric and electronic properties of 1–10

**Fig. 3:**
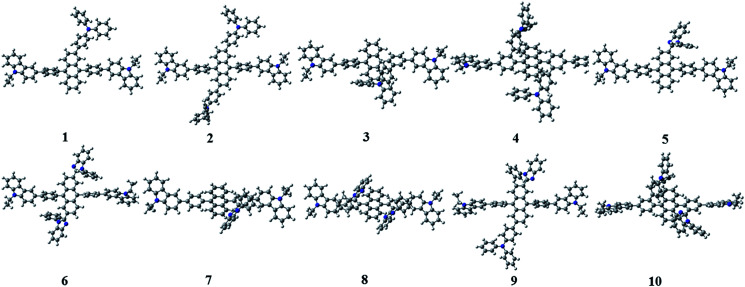
The optimized structures of 1–10.

**Fig. 4:**
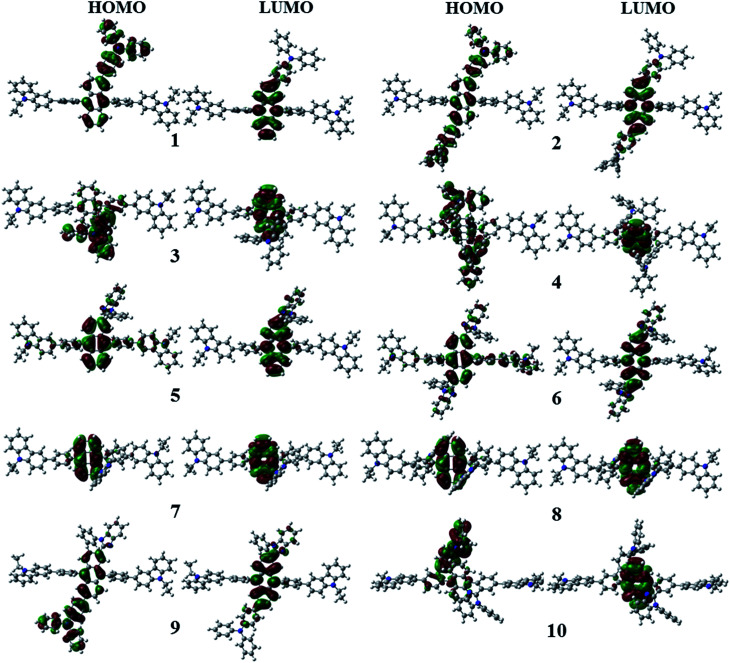
Electron density contour maps of HOMO and LUMO in 1–10.

Fig. 3 shows the optimized structures of 1–10. All optimized structures were verified as their global minimum energy points according to rotational potential energy surface simulations with the variation of the angles *θ*_Ph-Cz_, *θ*_Ph-TPA_, *θ*_Ph-PBI_, *θ*_Ant-TPA_, and *θ*_Ant-BPI_. The basic framework of 1–10 is the diphenyl anthracene core linked with ethyl-carbazole moieties. The additional substitutions of TPA and PBI were simultaneously or singly adopted in anthracene and bridging phenyl fragments to inhibit the structural relaxation *via* C–H in-plane bending vibration mode. As listed in Table 1, all bond lengths of 1–10 are similar. The maximum deviations do not exceed over 0.04 Å. It indicates that the additional substituents do not affect the bond lengths. On the other hand, the dihedral angles between bridging phenyl and anthracene fragments in the ground states are significantly increased or decreased, depending on the substituents. Interestingly, the dihedral angles of 1–10 in singlet excited states are tremendously reduced as compared with Cz3PhAn. It implies that effective conjugation can be relatively enhanced. The frontier molecular orbitals of 1–10 are shown in Fig. 4. As shown in Fig. 4, the electron density in HOMO of 1–4, 9, and 10 is predominantly distributed on TPA and anthracene core where the electron density contribution is more than 93%. For 5–8, the electron density in HOMO is mainly distributed over the anthracene and adjacent π-conjugated fragments with contribution of more than 90%. On the other hand, the electron density of LUMO in 1–10 is mostly localized on anthracene core with contribution over 75%. The vertical ionization potential (vIP) and vertical electron affinity (vEA) values can be used to characterize the reduction and oxidation ability, which can provide useful information for carrier injection property. The HOMO, LUMO, vIP, and vEA of all complexes were obtained from singlet point energy calculations with B3LYP/6-311G*, which is reported to give reliable results. As summarized in Table 2, the calculated HOMO/vIP and LUMO/vEA values are in excellent agreement with each other. As listed in Table 2, the vIP and vEA for Cz3PhAn are 5.52 and 2.02 eV, respectively. When the TPA and PBI fragments were substituted, the vIP values of 1–10 were generally reduced, while the vEA values are increased except for 7, 8, and 10. This indicates the injection barrier of holes and electrons from neighboring layers can be effectively lowered, resulting in the substantial improvement of hole and electron acceptability. Moreover, the chemical hardness values of 1–10 also demonstrate that the designed materials can effectively facilitate hole and electron transport as compared with Cz3PhAn.

Selected structural parameters of Cz3PhAn and 1–10 in ground and excited state

**Table:** 

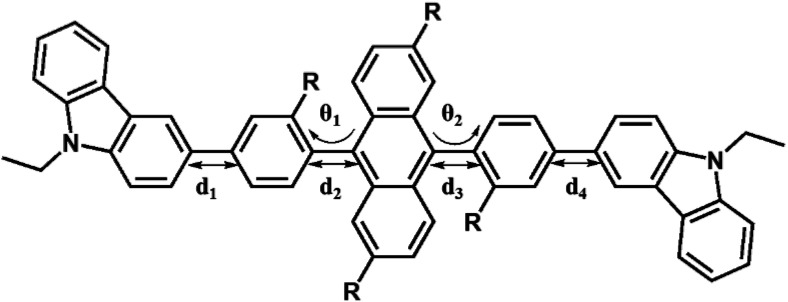												
	Cz3PhAn		1		2		3		4		5	
	S_0_	S_1_	S_0_	S_1_	S_0_	S_1_	S_0_	S_1_	S_0_	S_1_	S_0_	S_1_
*d*_1_	1.48	1.48	1.48	1.48	1.48	1.48	1.48	1.48	1.48	1.48	1.48	1.48
*d*_2_	1.49	1.47	1.49	1.48	1.49	1.48	1.50	1.48	1.50	1.48	1.49	1.47
*d*_3_	1.49	1.47	1.49	1.47	1.49	1.48	1.49	1.47	1.50	1.48	1.49	1.47
*d*_4_	1.48	1.48	1.48	1.48	1.48	1.48	1.48	1.48	1.48	1.48	1.48	1.48
*θ*_1_	82.90	55.51	88.66	60.04	83.13	59.37	84.63	54.31	75.83	64.95	78.29	57.42
*θ*_2_	82.85	55.48	78.67	53.87	82.60	58.94	105.47	118.03	77.92	67.92	76.69	56.20

**Table:** 

	6		7		8		9		10	
	S_0_	S_1_	S_0_	S_1_	S_0_	S_1_	S_0_	S_1_	S_0_	S_1_
*d*_1_	1.48	1.48	1.48	1.48	1.48	1.48	1.48	1.48	1.48	1.48
*d*_2_	1.49	1.47	1.50	1.48	1.50	1.48	1.49	1.47	1.50	1.48
*d*_3_	1.49	1.48	1.49	1.47	1.50	1.48	1.49	1.48	1.50	1.48
*d*_4_	1.48	1.48	1.48	1.48	1.48	1.48	1.48	1.48	1.48	1.48
*θ*_1_	74.34	55.97	92.76	127.17	84.74	69.82	−03.78	122.60	101.36	111.53
*θ*_2_	107.51	120.95	85.04	72.39	85.55	70.48	74.85	56.12	97.94	112.51

**Table:** HOMO and LUMO energy levels, vertical ionization potential (vIP), vertical electron affinity (vEA), and chemical hardness (*η*) of 1–10

Compounds	*E*_HOMO_	*E*_LUMO_	vIP	vEA	*η*
Cz3PhAn	−5.51	−2.03	5.52	2.02	1.75
1	−5.19	−2.09	5.17	2.10	1.54
2	−5.10	−2.16	5.08	2.18	1.45
3	−5.26	−2.05	5.25	2.04	1.61
4	−5.24	−2.07	5.22	2.05	1.59
5	−5.47	−2.22	5.45	2.23	1.61
6	−5.46	−2.38	5.43	2.40	1.52
7	−5.45	−2.00	5.46	1.98	1.74
8	−5.39	−1.97	5.40	1.95	1.73
9	−5.20	−2.27	5.18	2.30	1.44
10	−5.25	−2.02	5.25	2.00	1.63

### 3.3 Charge transport properties

**Fig. 5:**
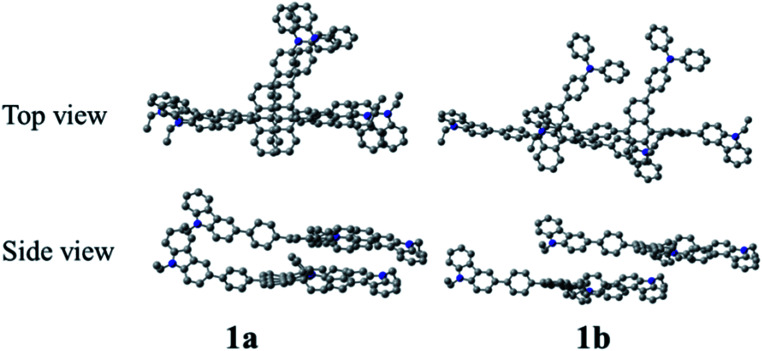
The dimer structures of co-facial and displaced-facial configurations. The hydrogen atoms are omitted for clarity.

In solid state, organic molecules can interact with neighboring molecules through non-covalent interaction, which is mainly originated from π–π and π–H interactions.^40–42^ Note that different types of dimer configurations for transfer integral should be considered such as co-facial and displaced-facial pair structures. Generally, it is well known that B3LYP functional is not proper to describe paired structures due to the lack of dispersive interaction.^43^ Therefore, ωB97XD functional including Grimmes's dispersion correction was adopted to predict the dimer structures.^44^ In addition, smaller 3-21G* basis set was employed due to computational cost. The transfer integrals (*t*_h_ and *t*_e_), reorganization energies (*λ*_h_ and *λ*_e_), and carrier hopping rates (*k*_h_ and *k*_e_) of 1–10 are listed in Table 3. In co-facial dimers, compared with Cz3PhAn, the *t*_h_ values show an improvement of orbital overlap, while *t*_e_ values are significantly reduced. On the other hand, in the case of displaced-facial dimers, both *t*_h_ and *t*_e_ values are mostly decreased except for 1 and 7. Considering the nature of organic bulk system, thermally evaporated organic molecules are in a similar shape of displaced-facial dimer configuration. Therefore, the carrier transport can be better understood on the basis of displaced-facial dimer configuration. From this perspective, the reduced *t*_h_ and *t*_e_ values in the derivatives can be interpreted that the addition of substituents may inhibit molecular orbital overlap (Fig. 5).

**Table:** Reorganization energy, transfer integral, and carrier hopping rates of 1–10

Compounds	Dimer	*λ*_h_	*λ*_e_	*t*_h_^2^	*t*_e_^2^	*k*_h_	*k*_e_
Cz3PhAn	Co-facial	0.402	0.384	2.02 × 10^−6^	1.86 × 10^−2^	1.07 × 10^9^	1.20 × 10^13^
	Displaced-facial			2.62 × 10^−5^	1.69 × 10^−4^	1.38 × 10^10^	1.09 × 10^11^
1	Co-facial	0.469	0.387	3.61 × 10^−3^	2.85 × 10^−3^	9.19 × 10^11^	1.78 × 10^12^
	Displaced-facial			2.27 × 10^−4^	1.02 × 10^−4^	5.77 × 10^10^	6.41 × 10^10^
2	Co-facial	0.460	0.409	8.84 × 10^−3^	1.69 × 10^−4^	2.49 × 10^12^	8.24 × 10^10^
	Displaced-facial			5.34 × 10^−7^	6.32 × 10^−7^	1.51 × 10^8^	3.08 × 10^8^
3	Co-facial	0.280	0.366	1.14 × 10^−4^	7.11 × 10^−3^	2.37 × 10^11^	5.61 × 10^12^
	Displaced-facial			5.53 × 10^−6^	1.46 × 10–^4^	1.15 × 10^10^	1.15 × 10^11^
4	Co-facial	0.259	0.320	2.54 × 10^−4^	5.31 × 10^−9^	6.71 × 10^11^	7.00 × 10^6^
	Displaced-facial			1.96 × 10^−5^	3.81 × 10^−5^	5.20 × 10^10^	5.01 × 10^10^
5	Co-facial	0.369	0.333	4.04 × 10^−5^	2.46 × 10^−4^	3.06 × 10^10^	2.80 × 10^11^
	Displaced-facial			9.23 × 10^−5^	8.98 × 10^−7^	6.99 × 10^10^	1.02 × 10^9^
6	Co-facial	0.305	0.304	1.62 × 10^−4^	4.49 × 10^−8^	2.51 × 10^11^	7.12 × 10^7^
	Displaced-facial			1.92 × 10^−5^	8.07 × 10^−7^	2.99 × 10^10^	1.28 × 10^9^
7	Co-facial	0.330	0.349	5.55 × 10^−3^	2.77 × 10^−3^	6.52 × 10^12^	2.62 × 10^12^
	Displaced-facial			1.81 × 10^−4^	1.26 × 10^−4^	2.12 × 10^11^	1.19 × 10^11^
8	Co-facial	0.360	0.336	1.95 × 10^−5^	8.23 × 10^−6^	1.64 × 10^10^	9.04 × 10^9^
	Displaced-facial			5.41 × 10^−9^	8.56 × 10^−9^	4.56 × 10^6^	9.39 × 10^6^
9	Co-facial	0.465	0.353	7.35 × 10^−4^	1.16 × 10^−5^	1.96 × 10^11^	1.05 × 10^10^
	Displaced-facial			8.04 × 10^−6^	2.86 × 10^−6^	2.14 × 10^9^	2.59 × 10^9^
10	Co-facial	0.397	0.317	1.64 × 10^−4^	5.84 × 10^−7^	9.17 × 10^10^	7.98 × 10^8^
	Displaced-facial			5.53 × 10^−6^	1.02 × 10^−9^	3.09 × 10^9^	1.40 × 10^6^

**Fig. 6:**
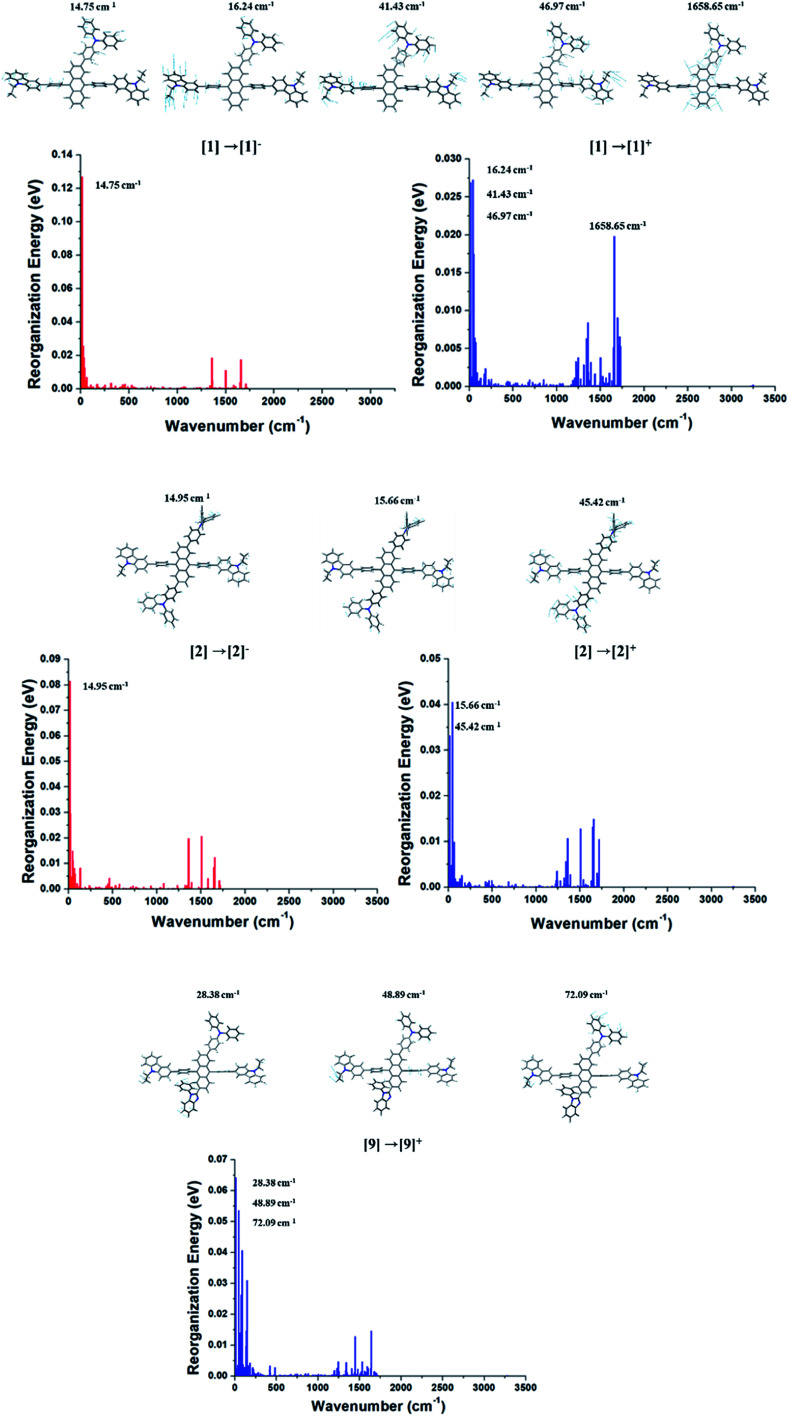
Energy contributions of vibration modes to *λ*_h_ and *λ*_e_ of 1, 2, and 9.

As aforementioned, the suppression of C–H in-plane bending of anthracene fragment might be the key to reduce the reorganization energy. The calculated *λ*_h_ and *λ*_e_ generally show that the reduced reorganization energies are attributed to additionally substituted TPA/PBI fragments. This result demonstrates that the modulation of specific vibration mode can effectively diminish the relaxation energy during charge transport (or migration). Nevertheless, the increased values of *λ*_h_ for 1, 2 and 9 and the increased *λ*_e_ for 1 and 2 were obtained. It is important to understand the origin of increased reorganization energies of 1, 2 and 9, which can be analyzed from the vibration modes coupled to the geometry change during the relaxation process. Therefore, these unexpected results of *λ*_h_ and *λ*_e_ during M^0^ → M^+^ and M^0^ → M^−^ were additionally analyzed in terms of Huang–Rhys factor. The results are shown in Fig. 6. In comparison with Cz3PhAn, the energy contributions of in-plane C–H bending modes around 1300–1700 cm^−1^ (at anthracene and bridging phenyl) are equally and sufficiently deactivated in *λ*_h_ and *λ*_e_ of 1, 2, and 9. On the other hand, the new vibration modes of 16.24, 41.43, and 46.97 cm^−1^, assigned as C–H out-of-plane bending modes are distinct from ethyl-carbazole and TPA fragments with high energy contributions on *λ*_h_ and *λ*_e_ of 1. In the case of 2, the new vibration modes of 15.66 and 45.42 cm^−1^ for M^0^ → M^+^ and 14.95 cm^−1^ for M^0^ → M^−^ originated from C–H out-of-plane bending mode dominantly contribute to the increase of *λ*_h_ and *λ*_e_. The new vibration modes of 2 are also arisen from ethyl-carbazole and TPA fragments. For 9, the mixed C–H out-of-plane and in-plane bending modes of 28.38, 48.89, and 72.08 cm^−1^ from ethyl-carbazole, TPA, and PBI fragments are strongly activated to increase of *λ*_h_. Interestingly, it is noticed that the substitution of TPA at 2 and 6 position of anthracene does not seem to have any benefit for reorganization energy. Note that the molecular rigidity of TPA substituent is relatively worse than that of PBI due to rotational degree of freedom. This result gives us an insight into a design strategy that the rigid fragments are essential to control the reorganization energy in terms of rotational energy. Based on the calculated *t*_h_, *t*_e_, *λ*_h_, and *λ*_e_, the *k*_h_, and *k*_e_ are derived from eqn (1). The *k*_h_ values of 1 and 3–7 are relatively larger than that of Cz3PhAn. On the other hand, the *k*_e_s are substantially smaller than that of Cz3PhAn except for 3 and 7. These results show that 7 out of 10 newly designed Cz3PhAn derivatives have relatively higher hole hopping rate than their parent molecule. It is also noteworthy that the ratio of *k*_h_ and *k*_e_ might be indicative of indirect prediction of charge carrier balance of the designed molecules as non-doped emitters. Good charge balance is undoubtedly essential to inhibit the polaron-induced non-radiative exciton decay and increase the exciton concentrations. Note that the ratio of *k*_h_ and *k*_e_ of Cz3PhAn is 0.126. Almost perfectly balanced hole and electron hopping rate were obtained in 1 and 4, whose charge balance can possibly be unity. Therefore, it is expected that the device efficiency with 1 or 4 as emitting material can be relatively improved due to the good charge balance. In addition, it is also expected that exciton–polaron quenching might be tremendously reduced and TTF probability might be enhanced in 1 and 4 non-doped emitting material.

### 3.4 Photo-physical properties

**Fig. 7:**
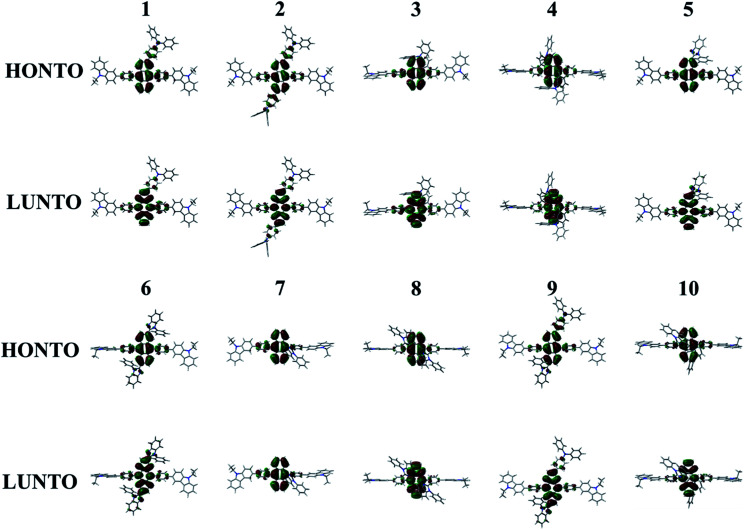
Natural transition orbitals of 1–10 for the transition to 1^st^ singlet excited state.

The absorption (*λ*_abs_) and emission (*λ*_em_) wavelengths, oscillator strengths (*f*), MO contributions of 1^st^ singlet excited state, and excited state lifetime of Cz3PhAn and 1–10 are listed in Table 4. The simulated *λ*_abs_ and *λ*_em_ values of Cz3PhAn (353.86 and 445.95 nm) are in good agreement with experimental results (359 and 440 nm). The absorption wavelengths of 1–10 are generally shifted to longer wavelengths as compared with Cz3PhAn. It can be interpreted that the additional substituents can lead destabilized HOMO and stabilized LUMO energy levels. Moreover, the electron density distributions are extended up to the bridging phenyl and additional substituents reinforce the effective conjugation by small torsion angles in Cz3PhAn derivatives. As shown in Table 4, the emission wavelengths of 1–10 are in the range of 439.11–473.6 nm. It is analyzed that HOMO → LUMO transition solely contributes to 1^st^ excited state transition in all the derivatives. The calculated emission wavelengths of 1–7 and 9 are shifted to longer wavelengths while those of 8 and 10 exhibit hypsochromic shift as compared with Cz3PhAn. To identify the transition characteristics, the natural transition orbitals (NTOs) were analyzed.^45^ As can be seen in Fig. 7, the hole- and electron-NTOs predominantly lie on the anthracene and partially distributed over TPA/PBI or bridging phenyl fragments, indicating that the transition characteristics is a typical π–π* transition. It is also noteworthy that the relaxation energy, which is obtained by subtracting emission energy from absorption energy, is relatively smaller than that of Cz3PhAn, except for 7. It implies that the non-radiative decay corresponding to the structural changes might be slightly suppressed in the Cz3PhAn derivatives. In fluorescence emitters, the short-excited state lifetime not only impacts on the suppression of the bimolecular annihilation such as singlet–singlet annihilation and singlet–polaron quenching effects, but also improves quantum efficiency. The excited state lifetimes of 1–10 were obtained using eqn (6). The calculated excited state lifetimes of 1–10 are slightly longer than that of Cz3PhAn since the oscillation strength of all complexes is substantially smaller than that of Cz3PhAn. Nevertheless, the differences of excited state lifetimes between the derivatives and the parent molecule are quite small. Thus, any side effects relevant to excited state lifetime such as decrease of efficiency and device lifetime are not expected. Considering the relaxation energies and excited state lifetimes, all 1–10 derivatives may show similar quantum efficiency.

**Table:** The photo-physical properties of 1–10

Compounds	*λ*_abs_ (nm)	*λ*_em_ (nm)	*E*_relaxation_	*f*	*E*_Flu_ (eV)	HOMO to LUMO contribution (S_1_ to S_0_)	*τ* (ns)
Cz3PhAn	353.86	445.90	0.724	0.7461	2.78	97%	2.08
1	366.58	464.30	0.712	0.4894	2.72	97%	2.97
2	379.39	473.63	0.650	0.4720	2.67	96%	3.11
3	359.60	452.39	0.707	0.5419	2.79	100%	2.52
4	364.93	447.93	0.630	0.4178	2.80	97%	3.16
5	366.37	464.26	0.714	0.5312	2.73	100%	2.73
6	379.43	468.88	0.623	0.4235	2.70	100%	3.32
7	354.75	447.90	0.727	0.5646	2.81	100%	2.40
8	356.21	439.11	0.657	0.4500	2.86	100%	2.90
9	382.31	473.25	0.623	0.4475	2.64	97%	3.26
10	360.58	443.17	0.641	0.4294	2.83	100%	3.06

## 4. Conclusions

A series of non-doped blue emitters were rationally designed based on Cz3PhAn. The electronic, photo-physical and charge transport properties of 1–10 are theoretically investigated by employing with DFT and TD-DFT calculations. Compared with Cz3PhAn, the designed 1–10 derivatives generally show unstable HOMO and stable LUMO energy levels, which can effectively facilitate the hole and electron injection from neighboring layers. Regarding charge transport property, the calculated hole hopping rates of 1, 4, and 5–7 are definitely improved due to effectively suppressed *λ*_h_. In addition, the ratio of hole and electron hopping rates of 1 and 4 showed excellent mobility balance, which may potentially result in good charge balance and superb device performance. The emission wavelength, relaxation energies, and excited state lifetimes of 1–10 are necessary and sufficient condition for blue fluorescent emitting materials. We expect that this study can provide a comprehensive insight to design blue emitting materials for non-doped fluorescent OLEDs with high efficiency. In particular, one of the most significant issues in commercial blue fluorescent device is that the device efficiency is almost saturated due to the theoretical limitation of singlet exciton consumption. Besides, the anthracene based TTF emitters are generally used in commercial blue fluorescent device. From this perspective, we also expect that our study can give a further driving force to overcome current limitation of commercial fluorescent device efficiency.

## Conflicts of interest

There are no conflicts of interest to declare.

## Supplementary Material

RA-009-C9RA04918E-s001

## References

[cit1] Tang C. W., VanSlyke S. A. (1987). Appl. Phys. Lett..

[cit2] Chien C. H., Chen C. K., Hsu F. M., Shu C. F., Chou P. T., Lai C. H. (2009). Adv. Funct. Mater..

[cit3] Jeon S. H., Cho Y. M., Kim T., Kang S. (2019). J. Mater. Sci..

[cit4] Lee S. J., Park J. S., Yoon K. J., Kim Y. I., Jin S. H., Kang S. K., Gal Y. S., Kang S., Lee J. Y., Kang J. W. (2008). Adv. Funct. Mater..

[cit5] Xing X., Zhang L., Liu R., Li S., Qu B., Chen Z., Sun W., Xiao L., Gong Q. (2012). ACS Appl. Mater. Interfaces.

[cit6] Kim T., Lee K. H., Lee J. Y. (2018). J. Mater. Chem. C.

[cit7] Liu Y., Wei X., Li Z., Liu J., Wang R., Hu X., Wang P., Yamada-Takamura Y., Qi T., Wang Y. (2018). ACS Appl. Energy Mater..

[cit8] Kim H. G., Kim K. H., Kim J. J. (2017). Adv. Mater..

[cit9] Liu H., Zeng J., Guo J., Nie H., Zhao Z., Tang B. Z. (2018). Angew. Chem., Int. Ed..

[cit10] Ekbote A., Han S. H., Jadhav T., Mobin S. M., Lee J. Y., Misra R. (2018). J. Mater. Chem. C.

[cit11] Zeng W., Zhao Y., Ning W., Gong S., Zhu Z., Zou Y., Lu Z.-H., Yang C. (2018). J. Mater. Chem. C.

[cit12] Keruckienė R., Volyniuk D., Bezvikonnyi O., Masimukku N., Ivaniuk K., Stakhira P., Gražulevičius J. V. (2018). Dyes Pigm..

[cit13] Islam A., Zhang D., Usman K., Siddique A. H., Wattoo A. G., Khalid H., Ouyang X., Duan L., Ge Z. (2018). Opt. Mater..

[cit14] Chang Y.-C., Yeh S.-C., Chen Y.-H., Chen C.-T., Lee R.-H., Jeng R.-J. (2013). Dyes Pigm..

[cit15] Kondakov D. Y. (2015). Philos. Trans. R. Soc., A.

[cit16] Chiang C. J., Kimyonok A., Etherington M. K., Griffiths G. C., Jankus V., Turksoy F., Monkman A. P. (2013). Adv. Funct. Mater..

[cit17] Luo Y., Aziz H. (2010). Adv. Funct. Mater..

[cit18] Aydemir M., Haykır G., Battal A., Jankus V., Sugunan S. K., Dias F. B., Al-Attar H., Türksoy F., Tavaslı M., Monkman A. P. (2016). Org. Electron..

[cit19] Xiang J., Chen Y., Jia W., Chen L., Lei Y., Zhang Q., Xiong Z. (2016). Org. Electron..

[cit20] Di D., Yang L., Richter J. M., Meraldi L., Altamimi R. M., Alyamani A. Y., Credgington D., Musselman K. P., MacManus-Driscoll J. L., Friend R. H. (2017). Adv. Mater..

[cit21] Kukhta N. A., Matulaitis T., Volyniuk D., Ivaniuk K., Turyk P., Stakhira P., Grazulevicius J. V., Monkman A. P. (2017). J. Phys. Chem. Lett..

[cit22] Hu D., Yao L., Yang B., Ma Y. (2015). Philos. Trans. R. Soc., A.

[cit23] Serevičius T., Komskis R., Adomėnas P., Adomėnienė O., Kreiza G., Jankauskas V., Kazlauskas K., Miasojedovas A. n., Jankus V., Monkman A. (2017). J. Phys. Chem. C.

[cit24] Northey T., Penfold T. (2018). Org. Electron..

[cit25] Luo Y.-J., Lu Z.-Y., Huang Y. (2016). Chin. Chem. Lett..

[cit26] Zhou J., Chen P., Wang X., Wang Y., Wang Y., Li F., Yang M., Huang Y., Yu J., Lu Z. (2014). Chem. Commun..

[cit27] Yanai T., Tew D. P., Handy N. C. (2004). Chem. Phys. Lett..

[cit28] Frisch M. , Trucks G., Schlegel H., Scuseria G., Robb M., Cheeseman J., Scalmani G., Barone V., Mennucci B., Petersson G., et al., Gaussian 09, Gaussian Inc., Wallingford CT, 2009

[cit29] Scalmani G., Frisch M. J. (2010). J. Chem. Phys..

[cit30] Cornil J., Brédas J. L., Zaumseil J., Sirringhaus H. (2007). Adv. Mater..

[cit31] Valeev E. F., Coropceanu V., da Silva Filho D. A., Salman S., Brédas J.-L. (2006). J. Am. Chem. Soc..

[cit32] Gruhn N. E., da Silva Filho D. A., Bill T. G., Malagoli M., Coropceanu V., Kahn A., Brédas J.-L. (2002). J. Am. Chem. Soc..

[cit33] Geng Y., Li H., Wu S., Duan Y., Su Z., Liao Y. (2011). Theor. Chem. Acc..

[cit34] Wang X., Chen C., Li Y., Ning P., Wu W., Wang L. (2017). Org. Electron..

[cit35] Lukeš V., Aquino A., Lischka H. (2005). J. Phys. Chem. A.

[cit36] Zhang Y., Zhang L., Wang R., Pan X. (2012). J. Mol. Graphics Modell..

[cit37] Wang Q., Aziz H. (2013). ACS Appl. Mater. Interfaces.

[cit38] Shinar J. (2012). Laser Photonics Rev..

[cit39] Luo Y., Aziz H., Xu G., Popovic Z. D. (2007). Chem. Mater..

[cit40] Parent A. A., Ess D. H., Katzenellenbogen J. A. (2014). J. Org. Chem..

[cit41] Mishra B. K., Deshmukh M. M., Venkatnarayan R. (2014). J. Org. Chem..

[cit42] Kim K. S., Tarakeshwar P., Lee J. Y. (2000). Chem. Rev..

[cit43] Ramalho J. P. P., Gomes J. R., Illas F. (2013). RSC Adv..

[cit44] Chai J.-D., Head-Gordon M. (2008). Phys. Chem. Chem. Phys..

[cit45] Foster J., Weinhold F. (1980). J. Am. Chem. Soc..

