# Supplementary material for: Design of efficient non-doped blue emitters: toward the improvement of charge transport
Source: RSC Adv. 2019 Sep 4;9(48):27807–16. doi: 10.1039/c9ra04918e (PMC9070764; doi:10.1039/c9ra04918e)
Supplement: RA-009-C9RA04918E-s001 [file RA-009-C9RA04918E-s001.pdf]

Electronic Supplementary Material (ESI) for RSC Advances.
This journal is © The Royal Society of Chemistry 2019

Supplementary Information

Table of Contents

Fig. S1 The investigation of rotational potential energy surface of **1-10**. The red arrow indicates the dihedral angles of optimized structures of **1-10**.

Table S1 The results of HOMO, LUMO, λ_{abs}, and λ_{emi} of **Cz3PhAn** depending on the DFT functionals and basis sets.

Table S2 The cartesian coordinates of optimized structures of **1-10**. The units are in Å.

Fig. S1

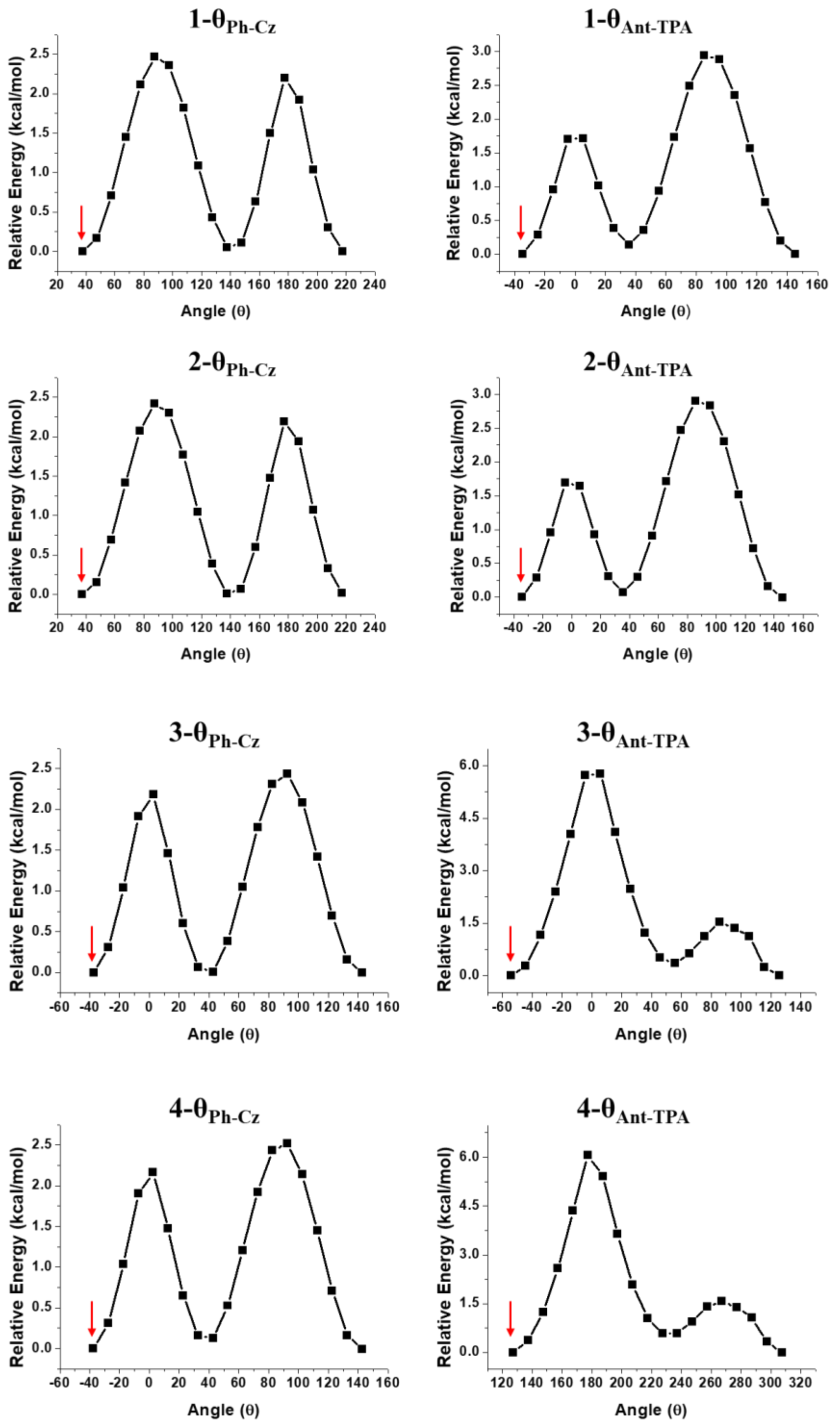
1-θPh-Cz
1-θAnt-TPA
2-θPh-Cz
2-θAnt-TPA
3-θPh-Cz
3-θAnt-TPA
4-θPh-Cz
4-θAnt-TPA
Relative Energy (kcal/mol)
Angle (θ)

5-θ_{Ph-Cz}

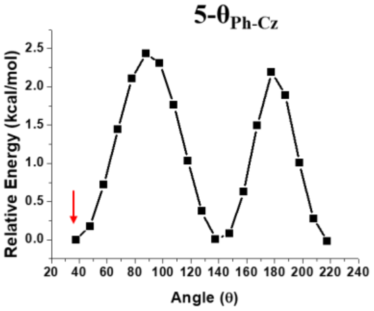

5-$\theta_{Ant-PBI}$

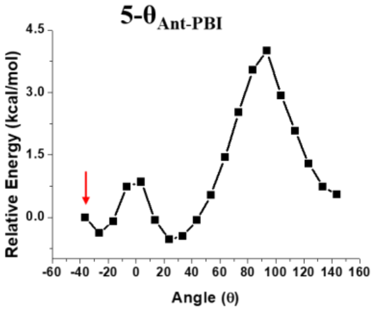

6-θ_{Ph-PBI}

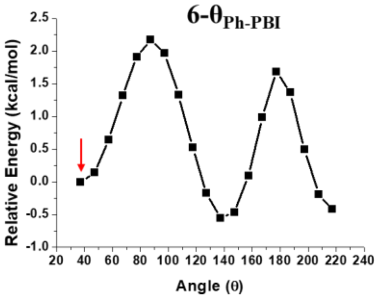

6-$\theta_{Ant-PBI}$

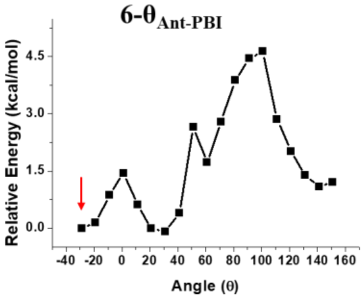

7-θ_{Ph-Cz}

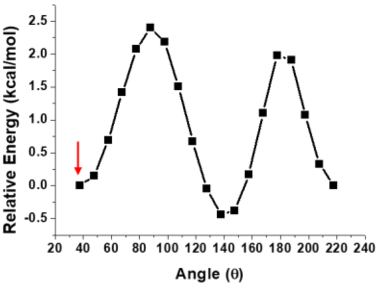

7-$\theta_{Ant-PBI}$

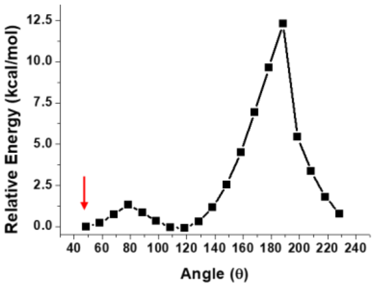

8-θ_{Ph-PBI}

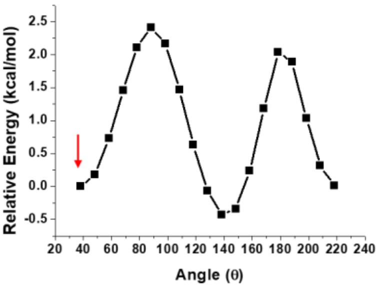

8-$\theta_{Ant-PBI}$

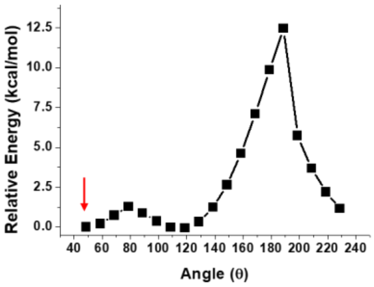

9-θ_{Ph-Cz}

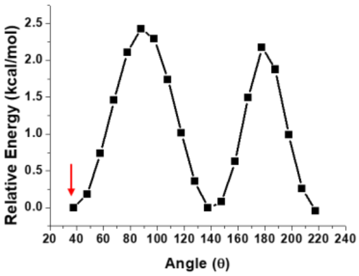

9-$\theta_{Ant-TPA}$

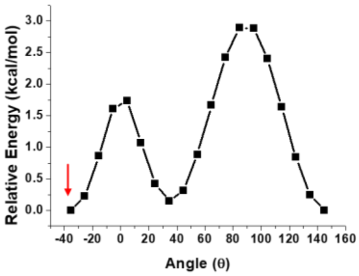

9-$\theta_{Ant-PBI}$

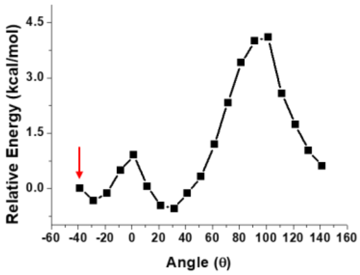

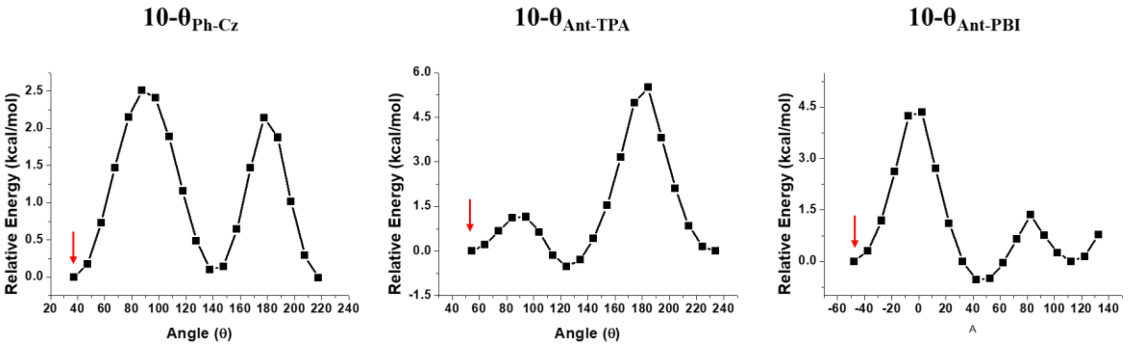

Table S1

	E_{HOMO} (eV)	E_{LUMO} (eV)	λ_{abs} (nm)	λ_{em} (nm)
Experiment	-5.52	-2.73	359	440
b3lyp/6-31G*	-4.99	-1.51	298.71	507.67
b3lyp/6-311G*	-5.51	-2.03	302.7	510.86
cam-b3lyp/6-31G*	-6.48	-0.61	353.86	445.9
cam-b3lyp/6-311G*	-6.75	-0.88	357.22	450.21

Table S2

1

C	-2.11265300	-5.68400900	0.21648300
C	-2.69323500	-4.45956000	0.08838200
C	-1.91028700	-3.26066900	0.06926200
C	-0.48293500	-3.37497900	0.18244300
C	0.08235300	-4.68386200	0.32339700
C	-0.70037200	-5.79737800	0.33910000
C	-2.50654100	-1.99336400	-0.05062900
C	0.32007100	-2.22099400	0.16617400
C	-0.27750300	-0.95378700	0.04399900
C	-1.70399500	-0.84224500	-0.06313000
C	-2.26486200	0.46980500	-0.18324400
H	-3.34194500	0.56870600	-0.24965500
C	-1.48245300	1.58028900	-0.20333800
C	-0.05747800	1.48517200	-0.11063000
C	0.50441600	0.24261400	0.00392700
H	-2.72634800	-6.57928500	0.22927800
H	-3.76993100	-4.37484900	-0.00007600
H	1.15752900	-4.77548400	0.42067000

H	-0.24835100	-6.77828500	0.44812000
H	1.58260600	0.14636700	0.04188500
C	-8.27626100	-1.50458900	-0.49201700
C	-8.98641600	-2.25501400	-1.45073300
C	-8.98231500	-0.64283700	0.34563400
C	-10.36113700	-2.16536800	-1.59190100
H	-8.44113400	-2.94583800	-2.08586000
C	-10.36513900	-0.53367800	0.22521600
H	-8.45172300	-0.04160900	1.07789600
C	-11.04944600	-1.29309300	-0.74864200
H	-10.87713200	-2.76868100	-2.33110000
C	-11.36957600	0.24790000	0.91518000
C	-12.60869300	-0.08348600	0.32385600
C	-11.32596800	1.17452900	1.95694500
C	-13.80172800	0.48999700	0.76484600
C	-12.50833800	1.75107500	2.39571000
H	-10.37910500	1.43924100	2.41849900
C	-13.73154000	1.40812700	1.80243800
H	-14.75570200	0.22941200	0.31892700
H	-12.48980100	2.47257400	3.20609300
H	-14.64623100	1.86840600	2.16324700
C	6.09232500	-2.67327200	0.61829000
C	6.81808700	-1.77496300	1.42637800
C	6.78407200	-3.66878200	-0.06976400
C	8.19428500	-1.84709100	1.56305600
H	6.28412900	-0.98250700	1.94095300
C	8.16808200	-3.76315900	0.04909000
H	6.24187900	-4.38309800	-0.68216200
C	8.86810900	-2.85336500	0.87095500
H	8.72265500	-1.12980200	2.18185600
C	9.16062700	-4.65476800	-0.51260400
C	10.40899700	-4.23396900	-0.00328700
C	9.09969900	-5.74309900	-1.38298200
C	11.59419300	-4.87877400	-0.35874100
C	10.27424300	-6.39039900	-1.73561200
H	8.14547000	-6.07729500	-1.77976200
C	11.50682000	-5.95778500	-1.22626400
H	12.55537100	-4.55122200	0.02295700
H	10.24219000	-7.23774900	-2.41281300
H	12.41521300	-6.47596800	-1.51783600
N	10.21782200	-3.15440400	0.84432200
N	-12.40078100	-1.00414600	-0.69070200
C	11.26756700	-2.40144800	1.50575600
C	11.82650200	-1.27088600	0.64764100
H	12.05938500	-3.10255600	1.78294400
H	10.85952800	-2.00971500	2.44128000
H	12.61077900	-0.73562400	1.19095000
H	12.25620800	-1.66143500	-0.27920400

H	11.04097600	-0.55641400	0.38555100
C	-13.43808600	-1.63527100	-1.48580200
C	-14.01091500	-2.89110700	-0.83653700
H	-13.01316100	-1.86757500	-2.46576600
H	-14.22560700	-0.89636500	-1.65738500
H	-14.78776300	-3.32508000	-1.47282600
H	-13.23025700	-3.64225200	-0.68614900
H	-14.45369100	-2.65912100	0.13627200
H	-1.94419300	2.55999200	-0.27069100
C	1.80430800	-2.33887400	0.28263300
C	2.45120100	-2.08923500	1.49471900
C	2.58520000	-2.69836200	-0.81761700
C	3.83121100	-2.19866700	1.60345900
H	1.86368900	-1.81588800	2.36624000
C	3.96580200	-2.80132200	-0.71014700
H	2.10520300	-2.88837800	-1.77304300
C	4.61748900	-2.55599600	0.50298400
H	4.30299900	-2.02471900	2.56551900
H	4.54932000	-3.05530800	-1.58956200
C	-3.99127300	-1.87071000	-0.16421800
C	-4.78676000	-1.71165200	0.97200500
C	-4.62119100	-1.91018600	-1.40942900
C	-6.16672100	-1.59806700	0.86642000
H	-4.31892300	-1.68650700	1.95173800
C	-6.00079500	-1.79156100	-1.51489100
H	-4.02150800	-2.02542200	-2.30746000
C	-6.80212400	-1.63355400	-0.37927500
H	-6.76239000	-1.50031100	1.76866500
H	-6.46065700	-1.80068900	-2.49817300
C	0.77694400	2.70845100	-0.14300000
C	0.41678900	3.81434600	-0.92037000
C	1.95945000	2.80074400	0.59950300
C	1.20627800	4.95287100	-0.97030600
H	-0.48035700	3.77492000	-1.53021300
C	2.74657300	3.94065300	0.56977200
H	2.25381400	1.97626800	1.24127200
C	2.38417900	5.03550100	-0.22173000
H	0.91319700	5.78500900	-1.60077200
H	3.64677900	3.99059200	1.17232000
N	3.18702700	6.19741600	-0.26111600
C	2.58273300	7.47971100	-0.32092700
C	4.60118500	6.08899800	-0.24948700
C	1.48558400	7.78569300	0.48898300
C	3.08039800	8.45572500	-1.18845800
C	5.36945800	6.95421400	0.53440600
C	5.24615000	5.12020400	-1.02351800
C	0.89405500	9.04070600	0.42188800
H	1.09937200	7.03479000	1.16988600

C	2.49518500	9.71462500	-1.23612600
H	3.92825300	8.22241400	-1.82355000
C	6.75498000	6.85504200	0.53510200
H	4.87471300	7.70454200	1.14171200
C	6.63105000	5.01509200	-1.00267300
H	4.65666600	4.45034000	-1.64016900
C	1.39670100	10.01396700	-0.43630500
H	0.04171600	9.26187800	1.05703800
H	2.89483300	10.46194100	-1.91481900
C	7.39448500	5.88320200	-0.22821200
H	7.33676300	7.53599700	1.14885100
H	7.11587500	4.25606800	-1.60900000
H	0.93703700	10.99596200	-0.48099900
H	8.47669400	5.80343600	-0.22003100

2

C	-2.43041800	-2.84661800	0.07034200
C	-2.42628600	-1.48070500	-0.01486200
C	-1.21925800	-0.71561400	-0.02047400
C	0.03304100	-1.40916600	0.07823900
C	-0.00135100	-2.83775400	0.16967000
C	-1.17380000	-3.52452200	0.16403900
C	-1.23842000	0.68851600	-0.10197500
C	1.24051100	-0.69315800	0.09177500
C	1.22136000	0.71103900	0.01062200
C	-0.03098900	1.40455600	-0.08775600
C	0.00327000	2.83322400	-0.17735700
H	-0.93492600	3.37123800	-0.24398500
C	1.17556400	3.52019400	-0.16933900
C	2.43241000	2.84249900	-0.07642200
C	2.42831700	1.47619200	0.00569300
H	-3.36705400	-0.94509000	-0.05459500
H	0.93682400	-3.37577000	0.23663000
H	-1.15963500	-4.60852300	0.21192700
H	3.36908800	0.94050700	0.04319500
C	-6.29704300	3.50767300	-0.53508500
C	-7.28670000	3.06119500	-1.43398100
C	-6.55502800	4.63387600	0.24464000
C	-8.50587200	3.70305900	-1.57246900
H	-7.09789800	2.16881200	-2.02209100
C	-7.77314200	5.29769900	0.12594300
H	-5.79817600	5.00574300	0.92882600
C	-8.74301900	4.83197700	-0.78825800
H	-9.25053100	3.32338100	-2.26369200
C	-8.34113300	6.46540000	0.76586700
C	-9.62587700	6.64201200	0.20628900
C	-7.88313100	7.35170900	1.74071200
C	-10.45937700	7.68437300	0.61307000
C	-8.70579000	8.39221200	2.14511200
H	-6.89661100	7.22690500	2.17760100
C	-9.98067500	8.55206600	1.58388100

H	-11.45032800	7.81611800	0.19176200
H	-8.36330800	9.08881100	2.90345400
H	-10.61049200	9.37124100	1.91704100
C	6.29955700	-3.51177800	0.52366500
C	7.28962700	-3.06471800	1.42182300
C	6.55726100	-4.63840800	-0.25554500
C	8.50888300	-3.70646200	1.56014900
H	7.10108000	-2.17200100	2.00950300
C	7.77547700	-5.30208300	-0.13704400
H	5.80010600	-5.01076400	-0.93912900
C	8.74572200	-4.83583200	0.77649700
H	9.25380600	-3.32636400	2.25085900
C	8.34323300	-6.47012400	-0.77656500
C	9.62819900	-6.64642300	-0.21740200
C	7.88484600	-7.35697200	-1.75074100
C	10.46153700	-7.68901400	-0.62392400
C	8.70735100	-8.39769500	-2.15489100
H	6.89814200	-7.23243300	-2.18729000
C	9.98245100	-8.55724400	-1.59406800
H	11.45263800	-7.82055200	-0.20290300
H	8.36456100	-9.09472600	-2.91269700
H	10.61212400	-9.37662100	-1.92700200
N	9.85431300	-5.66170000	0.73090700
N	-9.85160900	5.65783800	-0.74267200
C	11.08485000	-5.47134000	1.47666800
C	12.10369200	-4.60476500	0.74310600
H	11.50254400	-6.45860800	1.69205900
H	10.82565800	-5.02779800	2.44158400
H	13.00990400	-4.49449400	1.34589200
H	12.38079500	-5.05521700	-0.21424100
H	11.69784800	-3.60820800	0.54718600
C	-11.08202000	5.46764300	-1.48870000
C	-12.10088100	4.60069100	-0.75561600
H	-10.82262400	5.02449700	-2.45374400
H	-11.49976000	6.45496100	-1.70378700
H	-13.00698600	4.49053700	-1.35858400
H	-11.69498100	3.60411000	-0.55996300
H	-12.37818500	5.05076400	0.20184900
H	1.16106400	4.60428500	-0.21428500
C	2.54123300	-1.41936800	0.20088800
C	3.17808500	-1.56497400	1.43493900
C	3.15526600	-1.96951200	-0.92578000
C	4.38669600	-2.24055700	1.53902800
H	2.71461500	-1.15247800	2.32626000
C	4.36729400	-2.63949200	-0.82214600
H	2.68270800	-1.86100900	-1.89751900
C	5.00726000	-2.79073000	0.41242600
H	4.84489700	-2.36076300	2.51574800
H	4.83492700	-3.03303700	-1.71926100
C	-2.53910700	1.41476500	-0.21135900
C	-3.15401700	1.96383800	0.91535600
C	-3.17496600	1.56155100	-1.44577300
C	-4.36591100	2.63399700	0.81139700
H	-2.68223500	1.85437500	1.88736500
C	-4.38349200	2.23725700	-1.55017000
H	-2.71079400	1.14989700	-2.33711800

C	-5.00488700	2.78642800	-0.42354600
H	-4.83426300	3.02672000	1.70849600
H	-4.84095900	2.35837600	-2.52712500
C	3.69822900	3.61149300	-0.07742000
C	4.80685900	3.18843800	0.66439600
C	3.83402600	4.78570400	-0.82569400
C	5.99518000	3.90109100	0.66119800
H	4.72856800	2.30085600	1.28436100
C	5.02399500	5.49656400	-0.84884600
H	3.00628100	5.13538600	-1.43463300
C	6.12431000	5.06644300	-0.10138500
H	6.83065100	3.55986200	1.26245200
H	5.10660100	6.39048200	-1.45712000
N	7.33629000	5.79300100	-0.11318300
C	8.57856400	5.10879700	-0.11894900
C	7.31559300	7.21146700	-0.13115700
C	8.76456400	3.97998800	-0.92204900
C	9.63646200	5.55815600	0.67635200
C	8.17713600	7.92106100	-0.97202800
C	6.43625400	7.91827800	0.69373000
C	9.98203800	3.31128900	-0.91936600
H	7.95053800	3.62964200	-1.54742900
C	10.85694400	4.89517700	0.65884700
H	9.49691800	6.43010400	1.30623000
C	8.16267000	9.31009400	-0.97880300
H	8.85794000	7.37808800	-1.61877200
C	6.41463700	9.30690600	0.66751400
H	5.77010700	7.37349400	1.35411500
C	11.03688900	3.76603200	-0.13407600
H	10.10897600	2.43548500	-1.54838200
H	11.66869100	5.25872800	1.28155500
C	7.27947700	10.01170400	-0.16411500
H	8.83933600	9.84600700	-1.63739000
H	5.72452400	9.84056000	1.31386900
H	11.98931000	3.24597300	-0.14016200
H	7.26540900	11.09670200	-0.17692900
C	-3.69701200	-3.61556600	0.07544700
C	-3.83564200	-4.77936400	0.83920000
C	-4.80247000	-3.20199500	-0.67574700
C	-5.02080800	-5.49849700	0.85482700
H	-3.00500300	-5.12924700	1.44405400
C	-5.99710300	-3.90447000	-0.65316400
H	-4.72667500	-2.31442300	-1.29589800
C	-6.12400800	-5.06794800	0.11188800
H	-5.09734200	-6.39923500	1.45381200
H	-6.83926500	-3.55518200	-1.24023800
N	-7.33789700	-5.79279500	0.13302500
C	-7.80500500	-6.36188900	1.34540700
C	-8.09682300	-5.94714800	-1.05500500
C	-7.73480900	-5.64115400	2.54094900
C	-8.34490400	-7.65092700	1.36014800
C	-9.48746200	-5.80928400	-1.02937600
C	-7.46601100	-6.24271500	-2.26684600
C	-8.18635700	-6.20584600	3.72691900
H	-7.32434200	-4.63716200	2.53556300
C	-8.81214400	-8.20117000	2.54693300

H	-8.39682100	-8.21791000	0.43692600
C	-10.22943700	-5.97335700	-2.19214100
H	-9.98274600	-5.57424100	-0.09348800
C	-8.21328700	-6.38634700	-3.42887000
H	-6.38786000	-6.35860400	-2.29272600
C	-8.73167400	-7.48593600	3.73776500
H	-8.12315800	-5.63261900	4.64682700
H	-9.22964000	-9.20341900	2.54048300
C	-9.59851500	-6.25766000	-3.39926800
H	-11.30901400	-5.86422500	-2.15402000
H	-7.70713400	-6.61479200	-4.36186200
H	-9.09045500	-7.92156700	4.66464500
H	-10.18020600	-6.37812900	-4.30740500

3

C	0.02204800	-2.99511300	-3.36547500
C	0.79581900	-2.64911600	-2.30012400
C	0.21991600	-2.13913700	-1.09099400
C	-1.20620100	-1.97862000	-1.03436400
C	-1.98039800	-2.36324000	-2.17680900
C	-1.39172700	-2.85576600	-3.30063800
C	1.01412700	-1.77118000	0.00902600
C	-1.80936000	-1.44808700	0.11819500
C	-1.01866700	-1.10440500	1.22765600
C	0.40551300	-1.27867600	1.17696000
C	1.17092900	-0.93834300	2.33861300
H	2.24372000	-1.08745700	2.31361900
C	0.57876600	-0.44582700	3.46072300
C	-0.82947300	-0.25208500	3.50249900
C	-1.59721400	-0.57128500	2.42507800
H	0.48268400	-3.38004600	-4.26988900
H	1.87245600	-2.75866100	-2.35467900
H	-3.05747900	-2.25380200	-2.13134400
H	-1.99924600	-3.14107600	-4.15373500
H	-1.29011900	0.14809300	4.40028300
H	-2.67067000	-0.42842600	2.46127000
C	6.78611000	-2.42516600	-0.05863700
C	7.37071600	-3.55260400	-0.66994800
C	7.61554400	-1.47837200	0.54015300
C	8.74041500	-3.75477300	-0.69549000
H	6.72777700	-4.27702600	-1.15957100
C	8.99630100	-1.65644600	0.52771600
H	7.18403400	-0.61259400	1.03355200
C	9.55375800	-2.79898500	-0.08664600
H	9.15697900	-4.62699400	-1.18769600
C	10.10765000	-0.88671500	1.04551200
C	11.27852500	-1.60332700	0.71333500
C	10.20769500	0.31789100	1.74171400
C	12.54429000	-1.12872800	1.05857900
C	11.46314100	0.79142900	2.09166300
H	9.31455200	0.87732400	2.00426700
C	12.61689100	0.07157600	1.75029600
H	13.44551700	-1.67202900	0.79523700
H	11.55643600	1.72764900	2.63240300
H	13.59057000	0.46177200	2.03029300

C	-7.55280100	-0.60455600	0.27280300
C	-8.07951300	0.67958700	0.51813600
C	-8.43356300	-1.66525100	0.06749300
C	-9.44160400	0.92606200	0.56030500
H	-7.39501900	1.50088900	0.70480900
C	-9.80784200	-1.44525600	0.10496000
H	-8.04803400	-2.65855900	-0.14223200
C	-10.30691200	-0.14682000	0.34631500
H	-9.81266500	1.92426600	0.76643200
C	-10.96196900	-2.30211800	-0.06637400
C	-12.09801900	-1.47649600	0.08417600
C	-11.12491700	-3.66285100	-0.32622700
C	-13.39045400	-1.99326200	-0.01197600
C	-12.40735100	-4.18052400	-0.42749800
H	-10.25895100	-4.30735200	-0.44613000
C	-13.52564800	-3.34951800	-0.27037500
H	-14.26455200	-1.36313100	0.11321800
H	-12.54946300	-5.23748700	-0.62811800
H	-14.52114900	-3.77538100	-0.34987900
N	-11.68945400	-0.17307800	0.31788300
N	10.93020100	-2.76507400	0.04294500
C	-12.56418200	0.95344700	0.58666200
C	-12.90876300	1.10682300	2.06474200
H	-13.47159800	0.82253800	-0.00898200
H	-12.07393000	1.85496900	0.20965200
H	-13.56853700	1.96692100	2.21189600
H	-13.41825100	0.21492900	2.44045000
H	-12.00521600	1.26098000	2.66147100
C	11.85843300	-3.73140600	-0.51489900
C	12.30087600	-3.38879400	-1.93399600
H	11.37654200	-4.71230500	-0.48760800
H	12.72141700	-3.79202400	0.15372100
H	12.99707400	-4.14608200	-2.30622100
H	11.44252600	-3.34885200	-2.61063500
H	12.80302600	-2.41756900	-1.96162700
H	1.18043200	-0.19892800	4.32985200
C	-3.28752600	-1.23628600	0.15931500
C	-3.85378200	-0.06935300	-0.35825900
C	-4.13972400	-2.19156000	0.71556100
C	-5.22700800	0.13392600	-0.32213500
H	-3.21019900	0.68481800	-0.80214600
C	-5.51266100	-1.98542000	0.75592200
H	-3.72077600	-3.10316900	1.13130100
C	-6.08494900	-0.81979800	0.23626100
H	-5.64101800	1.03949800	-0.75476700
H	-6.14887500	-2.73440400	1.21705900
C	2.50246600	-1.90721000	-0.05473000
C	3.35175200	-0.79416500	-0.20559900
C	3.07908200	-3.17104100	0.08437700
C	4.73403100	-0.99066800	-0.20101400
C	4.45477500	-3.34823900	0.08419300
H	2.42931600	-4.03113500	0.21627300
C	5.31212200	-2.25383600	-0.05688100
H	5.37665500	-0.12861400	-0.35069000
H	4.86518900	-4.34346100	0.22190500
C	2.82082300	0.58831800	-0.35147200

C	3.24882900	1.60376800	0.50711700
C	1.88577900	0.91803500	-1.33679000
C	2.73203300	2.88908400	0.42024100
H	3.97143600	1.37473800	1.28477000
C	1.37978700	2.20343900	-1.44426800
H	1.55870700	0.16198500	-2.04206500
C	1.77931500	3.20195200	-0.55153300
H	3.05480800	3.65187700	1.12036100
H	0.66044100	2.43912400	-2.22099300
N	1.21461500	4.49848100	-0.62947800
C	2.03328500	5.64074800	-0.44934200
C	-0.18266300	4.64317000	-0.81991300
C	3.29721500	5.70341000	-1.04374200
C	1.59422000	6.71887800	0.32472700
C	-0.68361600	5.64490000	-1.65652300
C	-1.08095400	3.78492800	-0.17763900
C	4.10536700	6.81755600	-0.85791900
H	3.64180800	4.87369900	-1.65152700
C	2.40092600	7.83759200	0.48990100
H	0.61820900	6.67576600	0.79565000
C	-2.05340200	5.78779400	-1.83777300
H	0.00633300	6.31059600	-2.16379400
C	-2.44850800	3.92291200	-0.37929900
H	-0.70386100	3.00757200	0.47790400
C	3.66211900	7.89359200	-0.09501100
H	5.08443600	6.84823600	-1.32631100
H	2.04319000	8.66616000	1.09360400
C	-2.94481200	4.92620300	-1.20610200
H	-2.42390400	6.57209100	-2.49086700
H	-3.13017400	3.24640200	0.12791500
H	4.29262300	8.76608800	0.04210200
H	-4.01395300	5.03569100	-1.35626000

4

C	0.65912100	0.89051800	-3.62692400
C	1.29871000	0.48445700	-2.49636500
C	0.59887700	0.32261600	-1.25723100
C	-0.81020200	0.59904400	-1.22989600
C	-1.44286400	1.01329900	-2.44734100
C	-0.73711900	1.15760800	-3.60239000
C	1.25235100	-0.11991600	-0.09423700
C	-1.54050900	0.42335500	-0.04118000
C	-0.88068100	0.00957400	1.12901800
C	0.52581700	-0.27623700	1.09880800
C	1.15888300	-0.69894500	2.31314400
H	2.22079800	-0.91434000	2.29546500
C	0.45812900	-0.82552500	3.47335200
C	-0.93358700	-0.53592000	3.50420900
C	-1.57519500	-0.13397200	2.37325000
H	1.21186300	1.00472000	-4.55411200
H	2.36023900	0.26970100	-2.52287100
H	-2.50780500	1.21227500	-2.43603100
H	-1.23996700	1.47292700	-4.51126600
H	-1.48166500	-0.63213800	4.43617600
H	-2.63377500	0.09450900	2.40422100

C	6.81047800	-1.77381800	-0.42885300
C	7.16609500	-2.87049500	-1.23967500
C	7.80919600	-1.10840900	0.28008300
C	8.47342000	-3.31206700	-1.35643100
H	6.39272800	-3.37266000	-1.81210800
C	9.13182400	-1.53167400	0.18052100
H	7.55298700	-0.27395400	0.92598100
C	9.45808900	-2.63735400	-0.63459100
H	8.71434100	-4.15013400	-2.00159300
C	10.37332300	-1.07718600	0.77020700
C	11.38512600	-1.93063400	0.27750100
C	10.70487700	-0.04584400	1.64898300
C	12.72088300	-1.76152000	0.64337300
C	12.03032300	0.12338100	2.01958100
H	9.93539500	0.61599400	2.03564300
C	13.02460500	-0.72857300	1.51816000
H	13.50150300	-2.40927300	0.25891300
H	12.30332300	0.92213600	2.70153900
H	14.05698200	-0.57680900	1.81795900
C	-7.22841700	1.58742100	0.16970100
C	-7.69399200	2.68945800	0.91496700
C	-8.15295400	0.79219600	-0.50539400
C	-9.03819900	3.01107000	1.00032400
H	-6.97741800	3.29432900	1.46139100
C	-9.51055200	1.09299100	-0.43590700
H	-7.81286400	-0.04911800	-1.10180900
C	-9.94788800	2.20635100	0.31423400
H	-9.36284700	3.85808200	1.59505100
C	-10.69769200	0.49046500	-1.00447500
C	-11.79042300	1.26956200	-0.56441500
C	-10.92200800	-0.61446000	-1.82580500
C	-13.10075300	0.95424600	-0.92532600
C	-12.22202600	-0.92914000	-2.19160800
H	-10.08975200	-1.22013900	-2.17244400
C	-13.29729400	-0.14952800	-1.74235100
H	-13.94236900	1.54527200	-0.58005100
H	-12.41164500	-1.78656100	-2.82918300
H	-14.30783100	-0.41513400	-2.03726600
N	-11.32352100	2.31269500	0.21937800
N	10.81767000	-2.87909500	-0.55852000
C	-12.14819400	3.29020000	0.90552300
C	-12.59605200	2.83168000	2.28985000
H	-13.01309500	3.50278100	0.27137900
H	-11.57807700	4.22049100	0.97355100
H	-13.21407600	3.60127800	2.76153700
H	-13.18381000	1.91163600	2.22431200
H	-11.73354400	2.64074400	2.93470400
C	11.54166800	-3.89090000	-1.30590500
C	12.02167600	-3.40128800	-2.66857600
H	10.88545000	-4.75821400	-1.41710200
H	12.38671800	-4.21866600	-0.69448300
H	12.56046500	-4.19865600	-3.18879800
H	11.17688900	-3.09409100	-3.29161800
H	12.69471500	-2.54607100	-2.55999600
H	0.96124900	-1.14313400	4.38125900
C	-3.01376400	0.68139600	-0.01771300

C	-3.47127900	2.00058800	-0.00544400
C	-3.96178800	-0.35905400	0.03117700
C	-4.82352400	2.30315000	0.05242800
H	-2.74431000	2.80647400	-0.04246800
C	-5.31841400	-0.03517200	0.10177000
C	-5.77718100	1.28345700	0.10966500
H	-5.14028900	3.34107600	0.03573800
H	-6.03865700	-0.84563100	0.15616600
C	2.70154500	-0.48803200	-0.14319000
C	3.74054700	0.46023400	-0.15526500
C	3.03359600	-1.84278700	-0.22626100
C	5.06082800	0.01349700	-0.25832000
C	4.34995300	-2.26773100	-0.31716100
H	2.23397200	-2.57795400	-0.22256300
C	5.39453800	-1.33942300	-0.33617400
H	5.85358300	0.75472600	-0.28234100
H	4.56734800	-3.33047100	-0.35727800
C	3.49265800	1.92685600	-0.09495000
C	2.72494700	2.50889900	0.91741900
C	4.06283800	2.77615200	-1.04737300
C	2.52053600	3.87961200	0.96785000
H	2.27798500	1.88262200	1.68170700
C	3.87560800	4.14974600	-0.99712100
H	4.66612600	2.35380300	-1.84543200
C	3.09373400	4.72119300	0.00999700
H	1.91387200	4.30512400	1.75970700
H	4.33312400	4.78583200	-1.74694200
N	2.88744100	6.12050500	0.05872900
C	2.88619700	6.79502200	1.30593000
C	2.65930700	6.84710400	-1.13709700
C	3.84283700	6.48895600	2.27804000
C	1.92924900	7.77605900	1.58068300
C	3.25794600	8.09525800	-1.33189800
C	1.83155300	6.32821200	-2.13704600
C	3.83297100	7.14532500	3.50213400
H	4.59238500	5.73340100	2.06877200
C	1.93773800	8.44135300	2.80001800
H	1.17999700	8.01407100	0.83344000
C	3.02360400	8.81054900	-2.49926400
H	3.90647900	8.50149100	-0.56323200
C	1.61761300	7.04139300	-3.30962500
H	1.35834700	5.36322900	-1.99120000
C	2.88493400	8.12810000	3.76988500
H	4.58249400	6.89413900	4.24643900
H	1.18762300	9.20137100	2.99647900
C	2.20701700	8.28780700	-3.49726700
H	3.49581900	9.77904500	-2.63327500
H	0.97267900	6.62261400	-4.07614900
H	2.88440200	8.64438200	4.72438300
H	2.03193600	8.84580300	-4.41138100
C	-3.56516400	-1.79324100	0.05173000
C	-2.70306500	-2.33472800	-0.90629300
C	-4.05593600	-2.64662600	1.04365900
C	-2.32657500	-3.66767900	-0.86067700
H	-2.33231300	-1.70972300	-1.71097200
C	-3.67067300	-3.97849900	1.10681300

H	-4.72221900	-2.25193600	1.80493300
C	-2.78961000	-4.50282800	0.15931700
H	-1.66184100	-4.06936400	-1.61799000
H	-4.04023400	-4.61326600	1.90493900
N	-2.35619600	-5.85022900	0.23096100
C	-0.97861200	-6.14483200	0.07075100
C	-3.28257400	-6.88026500	0.52145300
C	-0.00620100	-5.32068400	0.64590100
C	-0.57202900	-7.25982200	-0.66786400
C	-2.93748200	-7.93345100	1.37433000
C	-4.56272100	-6.85733300	-0.04130800
C	1.34302200	-5.60539800	0.47630000
H	-0.31194000	-4.45657300	1.22578900
C	0.77881000	-7.54678800	-0.81827200
H	-1.32042100	-7.89986400	-1.12264000
C	-3.85123400	-8.94349400	1.64677600
H	-1.95022300	-7.95672100	1.82241700
C	-5.47626800	-7.86149800	0.25147400
H	-4.83713400	-6.04830000	-0.70950900
C	1.74499700	-6.72072300	-0.25246900
H	2.08426700	-4.95659600	0.93383500
H	1.07633600	-8.41733200	-1.39492400
C	-5.12700800	-8.91359900	1.09246000
H	-3.56460700	-9.75353700	2.31061900
H	-6.46567900	-7.82581600	-0.19438200
H	2.79955700	-6.94428500	-0.37711300
H	-5.84063700	-9.70067700	1.31333000

5

C	-1.22014300	-4.80084400	0.73618300
C	-1.93200400	-3.67356800	0.46194600
C	-1.28310600	-2.41671700	0.23841800
C	0.15000300	-2.36587000	0.31386400
C	0.85606400	-3.57662000	0.61126100
C	0.19852500	-4.75114600	0.81297800
C	-2.01539800	-1.24812100	-0.03728400
C	0.82488500	-1.14654600	0.11973600
C	0.09020900	0.02507200	-0.13596800
C	-1.34182300	-0.03124200	-0.22643700
C	-2.04572300	1.17701100	-0.53884600
H	-3.12454600	1.14035100	-0.62858100
C	-1.39352700	2.35192300	-0.72732000
C	0.02867800	2.42166000	-0.60780700
C	0.73759300	1.28605700	-0.33353100
H	-1.73446800	-5.74188300	0.90402900
H	-3.01346100	-3.71322600	0.41289000
H	1.93639800	-3.54270400	0.68075400
H	0.75594900	-5.65440200	1.04017100
H	1.81803100	1.32160000	-0.27649200
C	-7.80802700	-1.44122100	-0.39642000
C	-8.45111800	-2.51492200	-1.04455900
C	-8.58590700	-0.42462200	0.15529800
C	-9.82928300	-2.59525100	-1.15469700
H	-7.84784700	-3.32029200	-1.45121000
C	-9.97365900	-0.48056800	0.05869700

H	-8.10873600	0.42097700	0.64158600
C	-10.59037700	-1.56536500	-0.60178000
H	-10.29214000	-3.44348300	-1.64752900
C	-11.04225600	0.38786800	0.50550000
C	-12.24890700	-0.22057800	0.09488500
C	-11.07834100	1.59692100	1.20026600
C	-13.48736900	0.35637300	0.37755400
C	-12.30652900	2.17733200	1.47884600
H	-10.15710800	2.07588800	1.51903800
C	-13.49632400	1.55843600	1.06998700
H	-14.41570200	-0.11445800	0.07215400
H	-12.35013100	3.11733400	2.01931800
H	-14.44766100	2.02754400	1.30150000
C	6.62744500	-1.09094300	0.31533100
C	7.31910800	-0.02159500	0.91875800
C	7.36033300	-2.14312400	-0.23131900
C	8.70138900	0.02112100	0.99040800
H	6.74923200	0.81055300	1.31955900
C	8.75126800	-2.12539600	-0.17365000
H	6.84602000	-2.98573400	-0.68375000
C	9.41707000	-1.04378900	0.44309600
H	9.20179700	0.86721900	1.44906700
C	9.78171500	-3.03489600	-0.62823500
C	11.01627700	-2.45274800	-0.26537000
C	9.76370500	-4.25997300	-1.29509700
C	12.22932700	-3.07216300	-0.56771600
C	10.96645400	-4.88245900	-1.59298200
H	8.82055300	-4.71838300	-1.57789600
C	12.18446500	-4.28954200	-1.23141100
H	13.17907500	-2.62192900	-0.29933200
H	10.96796400	-5.83529800	-2.11231200
H	13.11512100	-4.79146600	-1.47765100
N	10.78263700	-1.25709500	0.39517900
N	-11.96249400	-1.39274500	-0.58652100
C	11.80152500	-0.33799300	0.86784000
C	12.23561100	0.67258200	-0.18919700
H	12.65505100	-0.93056300	1.20794300
H	11.40809500	0.17281100	1.75072600
H	13.00298200	1.33835500	0.21654500
H	12.64909500	0.16597500	-1.06587200
H	11.38865000	1.28297500	-0.51529600
C	-12.94067100	-2.32905600	-1.10890800
C	-13.37582300	-3.37508400	-0.08739900
H	-12.50729600	-2.80857600	-1.99064300
H	-13.80077000	-1.75317400	-1.46103300
H	-14.11048100	-4.05369600	-0.53066200
H	-12.52114600	-3.96765600	0.25123800
H	-13.83033900	-2.90044900	0.78688500
H	-1.93702300	3.25688700	-0.97271500
C	2.31648000	-1.11031100	0.17063200
C	2.99395700	-0.60294400	1.28041500
C	3.07405500	-1.60189800	-0.89534800
C	4.38202700	-0.59777000	1.32669800
H	2.42886100	-0.21290200	2.12165400
C	4.46112100	-1.58949300	-0.85114300
H	2.56826300	-1.99207400	-1.77350800

C	5.14446600	-1.09204900	0.26373400
H	4.87909900	-0.21978600	2.21417500
H	5.02324600	-1.95426100	-1.70512000
C	-3.50513600	-1.30078700	-0.12905900
C	-4.30512500	-0.86536500	0.92928200
C	-4.13723800	-1.78587800	-1.27569400
C	-5.69009900	-0.91580100	0.84449100
H	-3.83570800	-0.49182900	1.83453500
C	-5.52238300	-1.83075500	-1.36220800
H	-3.53563500	-2.12184600	-2.11504500
C	-6.32765400	-1.39722100	-0.30375100
H	-6.28645400	-0.59491300	1.69288600
H	-5.98532600	-2.18744600	-2.27697500
C	0.66731500	3.72407200	-0.85846200
N	0.23512700	4.55210600	-1.77733600
N	1.78128500	4.20064500	-0.18627600
C	1.08834600	5.63716800	-1.73210900
C	2.06400000	5.44300900	-0.74195000
C	2.41006200	3.68614500	0.98643400
C	1.08431500	6.81045400	-2.49112900
C	3.03680300	6.39886400	-0.45661000
C	1.65989000	3.44590200	2.13511500
C	3.78656700	3.47919900	0.98691100
C	2.05455700	7.76077700	-2.22334000
H	0.33473200	6.96275800	-3.26066000
C	3.01500400	7.55797100	-1.21633000
H	3.77616600	6.24700100	0.32164300
C	2.29209400	2.98465200	3.28307500
H	0.59055200	3.62553300	2.12599600
C	4.41461900	3.04025800	2.14746400
H	4.35744000	3.66074800	0.08259000
H	2.07592500	8.68223800	-2.79645900
H	3.75736900	8.32719800	-1.02875700
C	3.67020800	2.78761100	3.29458100
H	1.70658600	2.79531900	4.17684400
H	5.48999600	2.89392800	2.15097100
H	4.16232300	2.44165000	4.19777800

6

C	-1.13509000	3.65290700	-0.49522900
C	-1.69107900	2.42038000	-0.29640500
C	-0.88567000	1.24629100	-0.15886500
C	0.54127600	1.37823100	-0.24818300
C	1.08343200	2.68752500	-0.45837900
C	0.28459600	3.77748900	-0.58529500
C	-1.46376100	-0.01971700	0.05017500
C	1.36766900	0.24780700	-0.12572000
C	0.78927700	-1.01563300	0.09902600
C	-0.63662200	-1.14550800	0.19639200
C	-1.17530500	-2.44578600	0.46649900
H	-2.24838300	-2.55287300	0.56550800
C	-0.37612100	-3.53286500	0.60871100
C	1.04212200	-3.41824100	0.47338000
C	1.59373900	-2.19138400	0.22874500
H	-2.76717900	2.31191700	-0.25038800

H	2.15866400	2.80232400	-0.51414400
H	0.70900800	4.76087100	-0.75120400
H	2.66664300	-2.08343000	0.14894500
C	-7.22168200	-0.73476000	0.24792200
C	-8.04774700	0.22089700	0.87286900
C	-7.80956100	-1.86467400	-0.31834900
C	-9.42310700	0.07716900	0.94661700
H	-7.59283800	1.11385500	1.28956400
C	-9.19017000	-2.03400800	-0.25897000
H	-7.18929900	-2.62279800	-0.78704700
C	-9.99213300	-1.06314800	0.37944600
H	-10.03031100	0.83990300	1.42226100
C	-10.09168600	-3.06380500	-0.73050600
C	-11.39165300	-2.65898400	-0.35492000
C	-9.91257600	-4.26169100	-1.42244800
C	-12.51203100	-3.42903900	-0.66885700
C	-11.02237700	-5.03302600	-1.73251900
H	-8.91761900	-4.58430100	-1.71508700
C	-12.30731000	-4.61571300	-1.35755900
H	-13.51263900	-3.11591200	-0.39044600
H	-10.89835300	-5.96671200	-2.27144500
H	-13.16349700	-5.23244600	-1.61305500
C	7.12877600	0.83019400	-0.56266000
C	7.81051300	0.35328200	-1.70090900
C	7.86321800	1.43918900	0.45373700
C	9.18335900	0.46677800	-1.84533200
H	7.24374600	-0.13844400	-2.48514700
C	9.24442300	1.56586500	0.33444400
H	7.35627400	1.83332000	1.32933400
C	9.89993200	1.08213000	-0.81897800
H	9.67586200	0.07675800	-2.72956200
C	10.27191200	2.13280600	1.18197900
C	11.49475300	1.95823900	0.49744300
C	10.26034100	2.75579300	2.43006200
C	12.70341500	2.38828200	1.04591700
C	11.45824500	3.18947500	2.97741000
H	9.32609300	2.89754100	2.96554400
C	12.66517300	3.00452900	2.28812900
H	13.64504200	2.24675100	0.52618600
H	11.46473100	3.67479700	3.94797900
H	13.59247000	3.34835100	2.73597600
N	11.25550700	1.33560100	-0.71740700
N	-11.31715900	-1.45576900	0.32892500
C	12.26940400	0.92810200	-1.67274500
C	12.83389700	-0.46115300	-1.39286500
H	13.06513700	1.67767500	-1.65654900
H	11.82521800	0.97003700	-2.67072800
H	13.59155200	-0.71864500	-2.13873600
H	13.29851900	-0.50060200	-0.40355900
H	12.04438500	-1.21715900	-1.42983700
C	-12.44645100	-0.68723100	0.81931500
C	-13.00056100	0.29015100	-0.21260700
H	-12.12445100	-0.15619900	1.71919700
H	-13.21930500	-1.39251600	1.13594400
H	-13.84699800	0.84242700	0.20600500
H	-12.23605000	1.01109400	-0.51586000

H	-13.34390500	-0.23973200	-1.10563700
H	-0.79613300	-4.50711600	0.82849600
C	2.84920300	0.39183300	-0.23645500
C	3.45322200	0.64626200	-1.46959800
C	3.67056300	0.28406200	0.88790700
C	4.82946800	0.79339100	-1.57366200
H	2.83498000	0.74041700	-2.35730200
C	5.04815300	0.42011200	0.78091100
H	3.22510900	0.08222000	1.85754500
C	5.65665200	0.68079100	-0.45132300
H	5.26576100	1.01659200	-2.54207100
H	5.66218800	0.30497400	1.66851000
C	-2.94696000	-0.17968300	0.10343700
C	-3.63579600	-0.74174900	-0.97434700
C	-3.68226200	0.20811700	1.22462400
C	-5.01215400	-0.91367000	-0.93058300
H	-3.08528400	-1.04072800	-1.86134700
C	-5.05874600	0.02875800	1.27009100
H	-3.17151600	0.64903000	2.07541000
C	-5.75195600	-0.53752300	0.19570100
H	-5.52318600	-1.32916700	-1.79342300
H	-5.59939900	0.31707000	2.16560800
C	1.83994000	-4.63690800	0.68321100
N	1.44507500	-5.60088700	1.48052100
N	3.07838800	-4.88719900	0.11609800
C	2.45297900	-6.54349400	1.45570400
C	3.48695200	-6.11961900	0.60674700
C	3.76136400	-4.17666200	-0.91732200
C	2.55187200	-7.77051600	2.11810900
C	4.62507500	-6.88774600	0.37158100
C	3.18720000	-4.06329700	-2.18021200
C	5.01980700	-3.64090500	-0.65992000
C	3.68301900	-8.53759400	1.89837900
H	1.75800200	-8.10302200	2.77877800
C	4.70425100	-8.10232100	1.03432600
H	5.41155400	-6.55293200	-0.29567400
C	3.87517700	-3.39734800	-3.18691100
H	2.21049100	-4.49684700	-2.36634600
C	5.70760400	-2.98730400	-1.67617600
H	5.44901600	-3.73292600	0.33223200
H	3.78703800	-9.49507500	2.39884600
H	5.57508400	-8.73224900	0.88323300
C	5.13587200	-2.86279400	-2.93807200
H	3.42777700	-3.30460700	-4.17111400
H	6.68701300	-2.56547500	-1.47585100
H	5.67255600	-2.34934400	-3.72954800
C	-1.93795500	4.87311900	-0.67899500
N	-1.62228600	5.79636300	-1.55328200
N	-3.09909200	5.16589100	0.01776200
C	-2.60816900	6.75845300	-1.45083300
C	-3.54310400	6.38885000	-0.47143700
C	-3.64479400	4.51585000	1.16441700
C	-2.76088600	7.96086300	-2.14650900
C	-4.62778200	7.19573300	-0.13398400
C	-4.98617300	4.14401400	1.16348700
C	-2.85515900	4.30613700	2.29245400

C	-3.84275900	8.76317200	-1.82726600
H	-2.04322000	8.24856900	-2.90768800
C	-4.76093100	8.38625600	-0.83096400
H	-5.33492000	6.91008100	0.63649900
C	-5.54087900	3.57156000	2.30298500
H	-5.58716100	4.30292300	0.27455400
C	-3.41125900	3.71072500	3.41778400
H	-1.81546500	4.61407600	2.28584800
H	-3.98668000	9.70306700	-2.35061300
H	-5.59407600	9.04298800	-0.60176800
C	-4.75565800	3.34927000	3.42902200
H	-6.59089900	3.29715200	2.30657700
H	-2.79469300	3.54553600	4.29529400
H	-5.19010600	2.89889400	4.31560100

7

C	0.14899300	-0.78365500	3.29290000
C	-0.54553700	-0.85371900	2.12411000
C	0.11073100	-0.74494300	0.85507300
C	1.53860200	-0.59215000	0.84045000
C	2.22654100	-0.51370700	2.09504900
C	1.55979600	-0.60072300	3.27812200
C	-0.60265500	-0.79995700	-0.35278600
C	2.22586400	-0.53084300	-0.38259400
C	1.51393300	-0.62017200	-1.59126800
C	0.08490000	-0.75075400	-1.57616900
C	-0.60450000	-0.82226300	-2.82841600
H	-1.68484900	-0.90357700	-2.81971200
C	0.06745100	-0.78455300	-4.01107000
C	1.48489900	-0.66636500	-4.02671500
C	2.17957000	-0.58418900	-2.85933700
H	-0.37078200	-0.87506100	4.24162600
H	-1.61753900	-1.01157500	2.13956100
H	3.30244300	-0.38487100	2.08611600
H	2.10299500	-0.53938500	4.21583900
H	2.01056500	-0.63865900	-4.97613000
H	3.25900600	-0.49052600	-2.87440400
C	-6.34401100	-1.71087300	-0.22419500
C	-6.86271800	-2.84769500	0.42750800
C	-7.22632700	-0.82554500	-0.84101100
C	-8.22005700	-3.11748000	0.47634300
H	-6.17833200	-3.52396300	0.92971000
C	-8.59580800	-1.07257500	-0.80581900
H	-6.84523500	0.04446200	-1.36736600
C	-9.08742800	-2.22263700	-0.15048300
H	-8.58681600	-3.99439300	0.99896600
C	-9.75028700	-0.37647200	-1.33304800
C	-10.87929500	-1.14091300	-0.96496500
C	-9.91892400	0.79922400	-2.06459500
C	-12.17126500	-0.74216700	-1.30911700
C	-11.20053700	1.19730900	-2.41344600
H	-9.05822200	1.39437000	-2.35548300
C	-12.31233200	0.43083100	-2.03610100
H	-13.04086800	-1.32214200	-1.01899100
H	-11.34723100	2.11012600	-2.98163000

H	-13.30754800	0.76215000	-2.31620700
C	8.00423600	0.04559200	-0.45787100
C	8.61312100	1.04073100	-1.24889500
C	8.81297300	-0.79861200	0.30127300
C	9.98661200	1.21114300	-1.29778300
H	7.98635300	1.68109800	-1.86121800
C	10.19695600	-0.64998300	0.26949200
H	8.36283700	-1.55897900	0.93255900
C	10.77870700	0.35980700	-0.52738700
H	10.42247400	1.97773000	-1.92947600
C	11.29111500	-1.33938100	0.92005300
C	12.47704400	-0.71213800	0.47835800
C	11.36487200	-2.40763500	1.81399600
C	13.73191100	-1.14396700	0.90918700
C	12.60940300	-2.83712300	2.24944700
H	10.45977200	-2.89654700	2.16242600
C	13.77829400	-2.20841600	1.79760500
H	14.64483100	-0.67096600	0.56331400
H	12.68217000	-3.66784100	2.94388300
H	14.74308000	-2.56256500	2.14758900
N	12.15393500	0.32369700	-0.38363000
N	-10.46479900	-2.26240000	-0.26397000
C	13.10246100	1.15962900	-1.09626100
C	13.53252200	0.57103300	-2.43628800
H	13.96836700	1.31255300	-0.44660200
H	12.64347900	2.14192900	-1.23650000
H	14.24443000	1.23801700	-2.93130200
H	14.01227600	-0.40210700	-2.29823300
H	12.67101900	0.43690400	-3.09662000
C	-11.33606400	-3.25864100	0.33190000
C	-11.77327600	-2.90285500	1.74929600
H	-10.80741000	-4.21549700	0.32014300
H	-12.20477800	-3.37694900	-0.32116000
H	-12.42579800	-3.68363500	2.15090900
H	-10.90772400	-2.80471100	2.41066700
H	-12.32186000	-1.95676000	1.76176400
H	-0.47667400	-0.84057000	-4.94869900
C	3.71236400	-0.38122600	-0.39966900
C	4.30630300	0.88002400	-0.47321100
C	4.54601700	-1.49980700	-0.34590500
C	5.68816400	1.01790500	-0.48946200
H	3.67704400	1.76436200	-0.50866700
C	5.92751500	-1.36187800	-0.36929900
H	4.10573100	-2.49134600	-0.29686400
C	6.52741400	-0.10013000	-0.43935900
H	6.12155200	2.01267900	-0.52125300
H	6.55055000	-2.25070100	-0.35398100
C	-2.08841000	-0.97511400	-0.33033100
C	-2.99625500	0.08980600	-0.18968000
C	-2.60539000	-2.26840100	-0.42365000
C	-4.36724800	-0.17265200	-0.15045800
C	-3.96945000	-2.51584400	-0.38758600
H	-1.91424400	-3.09837500	-0.53350400
C	-4.88092600	-1.46596100	-0.25075600
H	-5.05559000	0.65681400	-0.03524600
H	-4.32944800	-3.53460600	-0.48909300

C	-2.54142800	1.49673900	-0.13824800
N	-1.70049200	2.02625600	-0.98904500
N	-3.03581000	2.42076000	0.77153100
C	-1.60902400	3.36052500	-0.64259200
C	-2.43827000	3.63159000	0.45646900
C	-3.89533800	2.21403800	1.89306100
C	-0.84926900	4.38544200	-1.21337900
C	-2.53231300	4.89788700	1.03009100
C	-5.15051400	2.81669700	1.90986200
C	-3.46393700	1.44933100	2.97219900
C	-0.94101500	5.64935600	-0.65612100
H	-0.20744500	4.18332800	-2.06453700
C	-1.77041500	5.90075000	0.45217200
H	-3.17098200	5.09022700	1.88521300
C	-5.98171800	2.64156700	3.01014800
H	-5.47019100	3.41157600	1.06045800
C	-4.30603300	1.26782400	4.06316200
H	-2.47281700	1.00981200	2.95448000
H	-0.36196000	6.46476000	-1.07795600
H	-1.81433900	6.90379300	0.86460200
C	-5.56338400	1.86304600	4.08472600
H	-6.96060700	3.10946800	3.02310100
H	-3.97295400	0.66695500	4.90318000
H	-6.21614000	1.72272200	4.94017500

8

C	0.82465100	-0.69795400	3.56055100
C	1.46256400	-0.33431600	2.41421600
C	0.75180600	-0.17418100	1.18055100
C	-0.66855000	-0.37791200	1.18485600
C	-1.29742100	-0.75645400	2.41397600
C	-0.58051800	-0.91993500	3.55873600
C	1.40716800	0.19016500	-0.00690200
C	-1.40721800	-0.19004900	0.00663000
C	-0.75185800	0.17430900	-1.18082000
C	0.66849900	0.37803300	-1.18512900
C	1.29737000	0.75657500	-2.41425100
H	2.37054900	0.90542300	-2.42011100
C	0.58046400	0.92007200	-3.55900700
C	-0.82471000	0.69812300	-3.56081300
C	-1.46262300	0.33448500	-2.41447700
H	1.38483100	-0.80826700	4.48401900
H	2.52914300	-0.14309100	2.42503900
H	-2.37059800	-0.90531500	2.41983200
H	-1.07993600	-1.20661900	4.47881300
H	-1.38489600	0.80846700	-4.48427200
H	-2.52920700	0.14328600	-2.42529800
C	7.10686000	1.32860100	0.14040400
C	7.60631300	2.29195100	1.03965500
C	7.99679100	0.67044100	-0.70649800
C	8.95228600	2.60892900	1.11169200
H	6.91716200	2.78600200	1.71708200
C	9.35533000	0.97018800	-0.65435900
H	7.62862000	-0.05948000	-1.42122600
C	9.82728500	1.94373900	0.25284500

H	9.30557200	3.34467700	1.82604400
C	10.51361600	0.48750900	-1.37611000
C	11.62526000	1.19226400	-0.86380500
C	10.69830600	-0.45769800	-2.38523000
C	12.91618100	0.95853600	-1.33874600
C	11.97867600	-0.69002100	-2.86420900
H	9.85098800	-1.00436500	-2.78878200
C	13.07332500	0.01346900	-2.34206000
H	13.77318000	1.49125200	-0.94051600
H	12.13781700	-1.42297800	-3.64846600
H	14.06808400	-0.18589200	-2.72859200
C	-7.10689300	-1.32855000	-0.14080800
C	-7.60637300	-2.29159200	-1.04037200
C	-7.99678700	-0.67070400	0.70637700
C	-8.95234600	-2.60856300	-1.11245200
H	-6.91724300	-2.78539500	-1.71800100
C	-9.35532300	-0.97046500	0.65421300
H	-7.62858400	0.05897300	1.42133800
C	-9.82730700	-1.94369800	-0.25331800
H	-9.30566000	-3.34406200	-1.82704900
C	-10.51357600	-0.48808300	1.37621400
C	-11.62523400	-1.19267900	0.86371800
C	-10.69822700	0.45673800	2.38570300
C	-12.91612900	-0.95917000	1.33883600
C	-11.97857000	0.68884100	2.86485700
H	-9.85089800	1.00327900	2.78940400
C	-13.07323300	-0.01448600	2.34251800
H	-13.77313900	-1.49176600	0.94046900
H	-12.13768100	1.42149600	3.64940200
H	-14.06797200	0.18469700	2.72919400
N	-11.19572100	-2.08054100	-0.10992200
N	11.19570900	2.08050000	0.10947800
C	-12.04999800	-2.93585600	-0.91328900
C	-12.56059200	-2.25530100	-2.17936400
H	-12.88478400	-3.25982200	-0.28607800
H	-11.48244700	-3.83653100	-1.16216600
H	-13.19744600	-2.94069300	-2.74624900
H	-13.14693900	-1.36522200	-1.93403200
H	-11.72832800	-1.95030600	-2.82014100
C	12.04996100	2.93609600	0.91257200
C	12.56052900	2.25598500	2.17889600
H	11.48239800	3.83685300	1.16112400
H	12.88476100	3.25985300	0.28526900
H	13.19736300	2.94157900	2.74555800
H	11.72825100	1.95120700	2.81975800
H	13.14688900	1.36582500	1.93389000
H	1.07988100	1.20675300	-4.47908500
C	-2.88410200	-0.42891700	-0.00343500
C	-3.34388000	-1.73144500	-0.20494800
C	-3.83923100	0.59247100	0.14703800
C	-4.69727900	-2.03019700	-0.25245200
H	-2.61567400	-2.52806300	-0.32178900
C	-5.19913500	0.27941100	0.09386700
C	-5.65518900	-1.02443600	-0.10236800
H	-5.01163000	-3.06023500	-0.38679900
H	-5.92469400	1.07739800	0.20424900

C	2.88405500	0.42901100	0.00314900
C	3.83916800	-0.59242700	-0.14708600
C	3.34385200	1.73157700	0.20437000
C	5.19907500	-0.27937900	-0.09395200
C	4.69725500	2.03031700	0.25182700
H	2.61565800	2.52823400	0.32101700
C	5.65515200	1.02450500	0.10199500
H	5.92461900	-1.07740700	-0.20413700
H	5.01161600	3.06038300	0.38593800
C	3.44409800	-1.99293100	-0.41468500
N	2.58891400	-2.35040100	-1.33793000
N	4.01517200	-3.07814000	0.23523600
C	2.56715800	-3.73155700	-1.31225700
C	3.45397600	-4.21015100	-0.33570200
C	4.90968600	-3.09712400	1.34831200
C	1.82599200	-4.63148200	-2.08312200
C	3.62444500	-5.56909100	-0.07959400
C	6.18875400	-3.62307900	1.18601700
C	4.48925400	-2.63110900	2.58987500
C	1.99342800	-5.98445800	-1.84232100
H	1.14055900	-4.26839200	-2.84180900
C	2.87970400	-6.44607000	-0.85194400
H	4.30717300	-5.92383300	0.68474400
C	7.05448900	-3.67065700	2.27251100
H	6.49942100	-3.98451000	0.21111300
C	5.36528500	-2.66962600	3.66842600
H	3.48046600	-2.25048500	2.70507400
H	1.43049800	-6.70659300	-2.42523500
H	2.98243700	-7.51421900	-0.68873400
C	6.64640800	-3.18929600	3.51250500
H	8.05200100	-4.07858800	2.14640200
H	5.04053300	-2.30103800	4.63604800
H	7.32590500	-3.22193500	4.35796900
C	-3.44419700	1.99293200	0.41492900
N	-2.58918700	2.35025700	1.33838900
N	-4.01515400	3.07824300	-0.23492600
C	-2.56745800	3.73141900	1.31296600
C	-3.45410700	4.21016700	0.33633200
C	-4.90945000	3.09739700	-1.34817000
C	-1.82646000	4.63122200	2.08413300
C	-3.62456400	5.56915000	0.08044200
C	-4.48871900	2.63176300	-2.58977600
C	-6.18862900	3.62310900	-1.18597400
C	-1.99388600	5.98423800	1.84354800
H	-1.14116200	4.26801200	2.84288500
C	-2.87998800	6.44600800	0.85309000
H	-4.30715000	5.92401400	-0.68396500
C	-5.36455600	2.67042600	-3.66847700
H	-3.47985300	2.25132100	-2.70489000
C	-7.05417000	3.67084000	-2.27261700
H	-6.49953800	3.98422800	-0.21103200
H	-1.43108200	6.70628000	2.42669900
H	-2.98271400	7.51418300	0.69005200
C	-6.64578600	3.18986600	-3.51266000
H	-5.03957000	2.30213600	-4.63613300
H	-8.05176800	4.07858500	-2.14658600

H	-7.32513200	3.22262100	-4.35824200

9

C	1.09533500	2.56193200	0.03780900
C	1.46147500	1.25008100	-0.09726900
C	0.50584100	0.18958600	-0.16107700
C	-0.88917700	0.51604200	-0.08474700
C	-1.24267300	1.89652200	0.05698300
C	-0.29900500	2.87249900	0.11345700
C	0.90621100	-1.15247600	-0.29270300
C	-1.85965300	-0.49965600	-0.12474300
C	-1.45738300	-1.84356300	-0.23458600
C	-0.06185800	-2.16798500	-0.33163200
C	0.30051700	-3.54477800	-0.49159200
H	1.34982700	-3.79758700	-0.58346700
C	-0.63574600	-4.52616600	-0.53546700
C	-2.02409100	-4.21333400	-0.40941800
C	-2.41080700	-2.90913400	-0.27561000
H	2.51080800	0.99397000	-0.17871600
H	-2.29055300	2.15906600	0.13657400
H	-0.60423700	3.90497200	0.24886200
H	-3.46208500	-2.65830000	-0.21378300
C	6.53649200	-2.51685900	-0.68492400
C	7.40077400	-1.76362800	-1.50469700
C	7.05384700	-3.60054000	0.02277000
C	8.74640300	-2.06363600	-1.63489500
H	7.00415000	-0.90401700	-2.03542200
C	8.40334400	-3.92426000	-0.09017600
H	6.39972400	-4.20509900	0.64395300
C	9.24410600	-3.15631300	-0.92496100
H	9.38635500	-1.45409500	-2.26374400
C	9.23422900	-4.96054700	0.48564400
C	10.53424700	-4.76193000	-0.02950100
C	8.99378400	-6.01207800	1.37013800
C	11.59521200	-5.59213800	0.33337000
C	10.04378700	-6.84329300	1.72997400
H	7.99804600	-6.17642000	1.77155200
C	11.33007500	-6.63050000	1.21426200
H	12.59650800	-5.43571400	-0.05359200
H	9.87162600	-7.66494200	2.41763900
H	12.13911900	-7.29071300	1.51137600
C	-7.46868300	0.97977000	0.11851000
C	-8.38707800	0.20963900	0.85992700
C	-7.92651000	2.11579700	-0.54690200
C	-9.72783500	0.54266100	0.95454400
H	-8.03642700	-0.69032100	1.35505100
C	-9.26978900	2.47354700	-0.46891500
H	-7.23151600	2.73374900	-1.10749000
C	-10.16549100	1.68725300	0.28817000
H	-10.40960600	-0.08107200	1.52253200
C	-10.04646100	3.56492500	-1.01796100
C	-11.37135000	3.37863200	-0.56570000
C	-9.74047900	4.65510800	-1.83272000
C	-12.39279400	4.25933100	-0.92272300
C	-10.75107900	5.53623200	-2.18650800

H	-8.72508400	4.81032500	-2.18566200
C	-12.06274600	5.33534700	-1.73371000
H	-13.41292000	4.11242000	-0.58425900
H	-10.52803500	6.38813400	-2.82064600
H	-12.83993000	6.03512600	-2.02522100
N	-11.42817000	2.24872700	0.23453000
N	10.52467800	-3.67760500	-0.89224900
C	-12.62800900	1.70132100	0.84025900
C	-13.38121500	0.74327100	-0.07727100
H	-13.26804400	2.53862400	1.13162900
H	-12.33600700	1.19862100	1.76603200
H	-14.27556600	0.36342900	0.42529100
H	-13.69223800	1.24780500	-0.99645100
H	-12.75279100	-0.10897100	-0.35124800
C	11.68268100	-3.12504300	-1.57048600
C	12.42504700	-2.08348100	-0.73925400
H	11.34252500	-2.69304800	-2.51541100
H	12.34603200	-3.95435400	-1.83043100
H	13.28813300	-1.70287700	-1.29326600
H	11.77211100	-1.23977200	-0.49827800
H	12.78307300	-2.51729200	0.19874500
H	-0.34897400	-5.56288700	-0.66787100
C	-3.30787600	-0.14279200	-0.06243800
C	-4.04874800	-0.32126200	1.10697200
C	-3.95522900	0.39792100	-1.17613900
C	-5.38841200	0.04003000	1.16359300
H	-3.57016900	-0.74135400	1.98653100
C	-5.29619900	0.75194300	-1.12053000
H	-3.40144500	0.53560500	-2.10004600
C	-6.03951000	0.58606700	0.05301500
H	-5.93049600	-0.08645400	2.09503800
H	-5.77879600	1.14627200	-2.00932500
C	2.35605400	-1.49735600	-0.39112400
C	3.06537700	-1.94510300	0.72504700
C	3.03900700	-1.38355600	-1.60366000
C	4.41280000	-2.26788000	0.63169700
H	2.55692200	-2.03286400	1.68064600
C	4.38507300	-1.71112800	-1.69749200
H	2.50573600	-1.04489500	-2.48696400
C	5.09969300	-2.16039600	-0.58206500
H	4.94501800	-2.59023900	1.52125900
H	4.88302100	-1.63859400	-2.65933400
C	-2.98142200	-5.32779400	-0.49965200
N	-2.80522400	-6.34018300	-1.31256800
N	-4.15724200	-5.42066500	0.22762800
C	-3.90489000	-7.15652600	-1.13480100
C	-4.76553800	-6.60449600	-0.17328600
C	-4.59528600	-4.62737400	1.32943700
C	-4.22519700	-8.37119400	-1.74686100
C	-5.93989600	-7.23856000	0.22731200
C	-3.76978000	-4.45075400	2.43726400
C	-5.87388300	-4.07742300	1.30790400
C	-5.39628400	-9.00254200	-1.36447100
H	-3.56504500	-8.79908000	-2.49409600
C	-6.23942300	-8.44434900	-0.38716000
H	-6.59011600	-6.81268400	0.98310500

C	-4.22503700	-3.70962900	3.52060600
H	-2.78156600	-4.89702700	2.44793200
C	-6.33029400	-3.35715600	2.40645500
H	-6.50274900	-4.21390300	0.43466800
H	-5.67080400	-9.94748800	-1.82242200
H	-7.14683600	-8.96964900	-0.10671200
C	-5.50730500	-3.16751400	3.51129400
H	-3.58029600	-3.57042300	4.38224500
H	-7.33286300	-2.94176100	2.39442900
H	-5.86455400	-2.60188900	4.36574000
C	2.10542500	3.64221400	0.11293900
C	1.86694700	4.89965600	-0.45178500
C	3.33567800	3.44411700	0.74938700
C	2.81630500	5.90850600	-0.40035400
H	0.93475400	5.08675200	-0.97566000
C	4.28386200	4.45180200	0.82217200
H	3.54254600	2.49293100	1.22966300
C	4.04051800	5.70171800	0.24303900
H	2.61208600	6.86537700	-0.86775900
H	5.21902000	4.27551300	1.34196600
N	5.00605200	6.73042100	0.31039700
C	4.60285000	8.07768400	0.49844700
C	6.38607700	6.42292900	0.19227400
C	3.61049100	8.39920300	1.42859200
C	5.19853700	9.10268500	-0.24146900
C	7.31945900	7.02663200	1.03919800
C	6.83172100	5.51758400	-0.77473400
C	3.21700200	9.71968800	1.60444600
H	3.14976700	7.60924400	2.01188600
C	4.81300500	10.42279700	-0.04618000
H	5.96556800	8.85882400	-0.96860500
C	8.67165000	6.73482300	0.91296100
H	6.97942700	7.72596600	1.79543000
C	8.18349200	5.21658500	-0.88209200
H	6.11347600	5.05107700	-1.44036500
C	3.81768000	10.73971400	0.87298400
H	2.44388400	9.95225900	2.33039900
H	5.28693600	11.20723200	-0.62829800
C	9.11215300	5.82547600	-0.04363500
H	9.38361500	7.21365400	1.57826800
H	8.51268200	4.51005300	-1.63790500
H	3.51336100	11.77117300	1.01818100
H	10.16837100	5.59387000	-0.13498300

10

C	-0.67493900	-1.25676900	-3.60367700
C	-1.45840800	-1.03799300	-2.51216000
C	-0.89301900	-0.83450500	-1.21072500
C	0.53638600	-0.87324400	-1.07892800
C	1.32080300	-1.09193900	-2.25784400
C	0.74125100	-1.27970800	-3.47528000
C	-1.69655500	-0.57134300	-0.08840900
C	1.13194200	-0.67884300	0.17710200
C	0.33128800	-0.40810800	1.29825100
C	-1.09655300	-0.35690200	1.16536200

C	-1.87666500	-0.09918500	2.33865400
H	-2.95596300	-0.07467500	2.24831300
C	-1.29080600	0.10738900	3.54951600
C	0.12477100	0.06279900	3.67861400
C	0.90344000	-0.19163100	2.59227500
H	-1.12850500	-1.40549300	-4.57849800
H	-2.53660800	-1.01081500	-2.61726800
H	2.40073400	-1.08589700	-2.16818100
H	1.35841100	-1.43568300	-4.35463900
H	0.58073100	0.22348500	4.65055000
H	1.98084000	-0.24016700	2.69570800
C	-7.49265800	-0.75253200	-0.43622000
C	-8.21079600	0.20893500	-1.17535600
C	-8.19648300	-1.76019400	0.22118100
C	-9.59237800	0.19013400	-1.26955900
H	-7.66204600	0.97742700	-1.71022800
C	-9.58586000	-1.80260900	0.14267100
H	-7.66279800	-2.49935800	0.81106400
C	-10.27952700	-0.82179600	-0.59908200
H	-10.11251400	0.93686100	-1.85986600
C	-10.59127400	-2.68657200	0.69344500
C	-11.83978300	-2.19492300	0.25250500
C	-10.54088100	-3.82368100	1.49977800
C	-13.03391600	-2.82838700	0.59820200
C	-11.72522700	-4.45397100	1.85038800
H	-9.58689400	-4.21029400	1.84636500
C	-12.95687900	-3.95735600	1.40070400
H	-13.99350100	-2.45888400	0.25249400
H	-11.70126200	-5.34018800	2.47623000
H	-13.87241500	-4.46779100	1.68347400
C	6.93096100	-0.54490800	0.56517900
C	7.62225100	-1.40708200	1.43954600
C	7.65911600	0.35700100	-0.20877500
C	9.00210700	-1.39183800	1.55551500
H	7.05424100	-2.08990200	2.06327100
C	9.04735300	0.39255700	-0.11191400
H	7.14573000	1.01695000	-0.90176100
C	9.71468100	-0.48771300	0.76771800
H	9.50139500	-2.05872500	2.25015200
C	10.07412300	1.18155200	-0.75897500
C	11.30784400	0.73850100	-0.23351300
C	10.05366600	2.19975300	-1.71223800
C	12.51684600	1.30375200	-0.64019200
C	11.25285200	2.76160000	-2.12338000
H	9.11144000	2.54814500	-2.12511600
C	12.46962900	2.31476900	-1.58887700
H	13.46487300	0.97180800	-0.23053400
H	11.25227600	3.55540100	-2.86330200
H	13.39706100	2.77068000	-1.92157800
N	11.07887500	-0.28102400	0.67691000
N	-11.63955600	-1.05868600	-0.51516000
C	12.09250600	-0.94488600	1.47611100
C	12.39746300	-0.21803200	2.78205200
H	12.99500300	-1.03531300	0.86551700
H	11.74859800	-1.96364400	1.67300600
H	13.16482200	-0.75756600	3.34488300

H	12.76243100	0.79458500	2.58810500
H	11.50166400	-0.14496000	3.40525600
C	-12.67494300	-0.30228600	-1.19501400
C	-12.98507900	-0.83011600	-2.59218900
H	-12.35159800	0.74107200	-1.24045100
H	-13.57037000	-0.32165900	-0.56796500
H	-13.76878200	-0.22633500	-3.05908800
H	-12.09656600	-0.79164600	-3.22887100
H	-13.33010200	-1.86709000	-2.54939400
H	-1.90369400	0.30109600	4.42433200
C	2.62233800	-0.70173100	0.30358600
C	3.37284700	-1.88124000	0.45815300
C	3.31198600	0.50997800	0.23465100
C	4.76516200	-1.80759300	0.53950200
C	4.69473200	0.57006400	0.31905700
H	2.74283300	1.42698000	0.11742500
C	5.45097000	-0.59439900	0.47290300
H	5.33167000	-2.72488100	0.65250400
H	5.19041300	1.53490600	0.28565600
C	-3.18710700	-0.56100800	-0.21655700
C	-3.85633100	-1.78475600	-0.29533400
C	-3.94919000	0.62193400	-0.21749300
C	-5.23871900	-1.85748000	-0.37201000
H	-3.27188100	-2.69998000	-0.29497500
C	-5.34191500	0.52861400	-0.28310000
C	-6.01165600	-0.69341800	-0.36248400
H	-5.72013700	-2.82656500	-0.45609500
H	-5.91900800	1.44796800	-0.26630200
C	-3.33592800	1.97512200	-0.12166100
C	-2.33980300	2.40221100	-1.00391500
C	-3.77728300	2.88010300	0.84762300
C	-1.79430600	3.67391500	-0.91198300
H	-1.98215200	1.73108800	-1.77717500
C	-3.24757200	4.15898700	0.93834500
H	-4.55113700	2.57738900	1.54672000
C	-2.24097300	4.57158600	0.06201900
H	-1.01654100	3.97859700	-1.60371500
H	-3.61082500	4.84293300	1.69763400
N	-1.68609300	5.87042500	0.15945800
C	-1.39511300	6.60794000	-1.01546200
C	-1.40385800	6.42646200	1.43266500
C	-2.29247500	6.61643800	-2.08735700
C	-0.20751200	7.33798000	-1.11980000
C	-1.68936100	7.76885800	1.69832100
C	-0.83534700	5.64144800	2.44003500
C	-2.00014700	7.33259200	-3.24104400
H	-3.21922800	6.05800300	-2.01125600
C	0.06865700	8.06681000	-2.26957000
H	0.49696200	7.33154200	-0.29514800
C	-1.40155900	8.31383200	2.94312500
H	-2.13674800	8.38218000	0.92359600
C	-0.56809500	6.18930800	3.68815700
H	-0.60537200	4.60048600	2.24023400
C	-0.82152200	8.06569200	-3.33894300
H	-2.70819500	7.32672100	-4.06417800
H	0.99546300	8.62890800	-2.33283900

C	-0.84396900	7.52832400	3.94723200
H	-1.62879900	9.35871900	3.13156300
H	-0.12732200	5.56473900	4.45920500
H	-0.59935100	8.63021500	-4.23869400
H	-0.62701400	7.95495400	4.92115800
C	2.72114200	-3.20271100	0.59156500
N	1.72093100	-3.44823100	1.39824800
N	3.16800500	-4.33940200	-0.06801100
C	1.46802000	-4.80099400	1.27945900
C	2.36340800	-5.38111100	0.36798500
C	4.16507900	-4.46268200	-1.08264500
C	0.50327900	-5.59146000	1.91021500
C	2.32760400	-6.73523900	0.04150400
C	5.30508700	-5.22086000	-0.82875200
C	3.97788200	-3.86115600	-2.32299500
C	0.46371100	-6.93974700	1.59878600
C	1.36333400	-7.50271500	0.67510100
C	6.26754400	-5.36679400	-1.82104800
H	5.43389300	-5.68455400	0.14385800
C	4.95126900	-4.00018700	-3.30553800
H	3.07102500	-3.29781000	-2.51305600
C	6.09509200	-4.75238300	-3.05739800
H	7.15669700	-5.95657000	-1.62368900
H	4.80910100	-3.52681000	-4.27159000
H	6.85059500	-4.86241500	-3.82853200
H	-0.19043800	-5.14915200	2.61759400
H	-0.27526000	-7.57818900	2.07252000
H	1.30042600	-8.56330600	0.45304600
H	3.02024900	-7.16843800	-0.67173800
